# Characterization of Pustular Mats and Related *Rivularia*-Rich Laminations in Oncoids From the Laguna Negra Lake (Argentina)

**DOI:** 10.3389/fmicb.2018.00996

**Published:** 2018-05-22

**Authors:** Estela C. Mlewski, Céline Pisapia, Fernando Gomez, Lena Lecourt, Eliana Soto Rueda, Karim Benzerara, Bénédicte Ménez, Stephan Borensztajn, Frédéric Jamme, Matthieu Réfrégiers, Emmanuelle Gérard

**Affiliations:** ^1^Centro de Investigaciones en Ciencias de la Tierra (CICTERRA), Cordoba, Argentina; ^2^Institut de Physique du Globe de Paris, Sorbonne Paris Cité, Centre National de la Recherche Scientifique, Université Paris Diderot, Paris, France; ^3^Synchrotron SOLEIL, DISCO Beamline, Saint Aubin, France; ^4^Institut de Minéralogie, de Physique des Matériaux et de Cosmochimie, UMR Centre National de la Recherche Scientifique 7590, Sorbonne Université, Muséum National d'Histoire Naturelle, IRD UMR 206, Paris, France

**Keywords:** *Rivularia*, carbonate precipitation, oncoids, Andean lakes, pustular mats

## Abstract

Stromatolites are organo-sedimentary structures that represent some of the oldest records of the early biosphere on Earth. Cyanobacteria are considered as a main component of the microbial mats that are supposed to produce stromatolite-like structures. Understanding the role of cyanobacteria and associated microorganisms on the mineralization processes is critical to better understand what can be preserved in the laminated structure of stromatolites. Laguna Negra (Catamarca, Argentina), a high-altitude hypersaline lake where stromatolites are currently formed, is considered as an analog environment of early Earth. This study aimed at characterizing carbonate precipitation within microbial mats and associated oncoids in Laguna Negra. In particular, we focused on carbonated black pustular mats. By combining Confocal Laser Scanning Microscopy, Scanning Electron Microscopy, Laser Microdissection and Whole Genome Amplification, Cloning and Sanger sequencing, and Focused Ion Beam milling for Transmission Electron Microscopy, we showed that carbonate precipitation did not directly initiate on the sheaths of cyanobacterial *Rivularia*, which dominate in the mat. It occurred via organo-mineralization processes within a large EPS matrix excreted by the diverse microbial consortium associated with *Rivularia* where diatoms and anoxygenic phototrophic bacteria were particularly abundant. By structuring a large microbial consortium, *Rivularia* should then favor the formation of organic-rich laminations of carbonates that can be preserved in stromatolites. By using Fourier Transform Infrared spectroscopy and Synchrotron-based deep UV fluorescence imaging, we compared laminations rich in structures resembling *Rivularia* to putatively chemically-precipitated laminations in oncoids associated with the mats. We showed that they presented a different mineralogy jointly with a higher content in organic remnants, hence providing some criteria of biogenicity to be searched for in the fossil record.

## Introduction

Microbialites record microbial activities and sedimentary processes under a lithified form (Walter et al., 1972; Burne and Moore, 1987). Stromatolites are laminated microbialites probably produced by lithification of microbial mats (Knoll, 2003; Bosak et al., 2007). Yet, comparable stromatolite-like products can form abiotically, casting doubts on the biological origin of some fossil stromatolites (Buick et al., 1981; Grotzinger and Knoll, 1999). Stromatolites are pervasive in the Precambrian fossil record. They can be traced back to the early Archean, 3.43 billion years (Awramik and Sprinkle, 1999; Allwood et al., 2006) and even 3.7 billion years ago (Nutman et al., 2016). Understanding the mechanisms of stromatolite formation is thus mandatory for the quest of the oldest traces of life on Earth and for documenting the early Earth environment. Notably, it is important to decipher the role played by microbial activity on the lithification processes. Microbial metabolisms known to favor carbonate precipitation (by reaching supersaturation with respect to carbonates through increased alkalinity) include oxygenic and anoxygenic photosynthesis, ureolysis, ammonification, denitrification, sulfate and iron reduction, anaerobic sulfide oxidation, or methane oxidation (Dupraz and Visscher, 2005; Dupraz et al., 2009; Zhu and Dittrich, 2016). In addition, microbial exopolymeric substances (EPS), consisting of a mixture of carbohydrates, proteins and nucleic acids, may promote or inhibit solid carbonate formation (e.g., Benzerara et al., 2006), possibly depending on their divalent-cation binding capacity (Braissant et al., 2007; Glunk et al., 2011). Among other phyla, cyanobacteria are important producers of EPS in microbialites (e.g., Foster et al., 2009) and it has long been suggested that the formation of some ancient stromatolites was mediated by oxygenic photosynthesis performed by cyanobacteria (Arp et al., 2001; Aloisi et al., 2006; Altermann et al., 2006; Riding, 2006). However, several studies have indicated that primary production in stromatolites could be partly performed by other organisms such as diatoms, anoxygenic phototrophs and/or non-phototrophic carbon fixers (Braissant et al., 2003; Bosak et al., 2007; Meister, 2013; Saghaï et al., 2015). The study of modern stromatolites may help to better understand the impact of these different metabolic processes on microbialite formation, some of which may have also played a role in the formation of ancient stromatolites. Furthermore, understanding stromatolites is attractive for petroleum geologists with the recent discovery of oil reservoir associated with stromatolites in the South Oman Salt Basin and the “pre-salt” deposits offshore of Brazil (Bosence et al., 2015). Overall, there is a need to document in a broader range of environments the type of microorganisms which contribute to carbonate precipitation in modern microbialites. Modern microbialites and stromatolites currently develop in restrictive, sometimes extreme environments such as the hypersaline Shark Bay in Australia (Logan, 1961), hot springs (Berelson et al., 2011), alkaline lakes (e.g., Kempe et al., 1991; Chagas et al., 2016) and also high-altitude lakes like Socompa, Brava, and Tebenquiche lakes in the Andes (Farías et al., 2013, 2014; Fernandez et al., 2015).

The Laguna Negra is a high-altitude hypersaline lake in Catamarca, Argentina where extreme environmental conditions (i.e., high UV-radiation and extreme temperature, salinity, and water activity) restrict eukaryotic life. Lithification is controlled by environmental processes (Gomez et al., 2014) along with microbial mats where diatoms and anoxygenic phototrophic bacteria are abundant (Gomez et al., 2018). This latter work focused on the texture, mineralogy and stable isotope geochemistry of subfossil oncoids and related microbialites from the Laguna Negra stromatolite belt. This belt mostly consists of carbonated laminar crusts, stromatolites and oncoids. Different types of carbonate laminations were identified within the oncoids based on their texture (e.g., micritic, sparry, botryoidal, tufted filament-rich palisade fabrics). These different laminations were partly associated with different microbial communities (i.e., stratified pink colored, greenish and black pustular mats; Gomez et al., 2018). Interestingly, laminations with calcified *Rivularia-*like cyanobacterial filaments showing tufted palisade fabrics were found alternating with micritic and botryoidal laminations. Consequently, potential changes in the microbial community could possibly be recorded within the oncoids as different types of laminations.

The main objectives of this study were (i) to investigate at Laguna Negra the potential role of a microbial consortium forming the black pustular mat (BP) on the initiation of carbonate precipitation and lamination formation and (ii) to identify biogenicity criteria for the corresponding laminations in subfossil oncoids. By jointly using Confocal Laser Scanning Microscopy (CLSM), Scanning and Transmission Electron Microscopy (SEM/TEM) associated with Energy Dispersive X-ray Spectrometry (EDXS) and Focused Ion Beam (FIB) milling, as well as phylogenetic analyses on the bulk BP mat and on laser microdissected mat cells, we highlighted the presence of an interesting microbial consortium associated with *Rivularia* filaments that triggers carbonate precipitation in the BP mats. In parallel, we determined the composition of the corresponding fossil laminations on oncoids associated with BP mats in order to identify potential biomarkers. By using Fourier Transform Infrared microspectroscopy (FTIR), powder X-Ray Diffraction (XRD) and Synchrotron-based deep UV fluorescence imaging (S-DUV), we clearly highlighted that these laminations present a different pattern compared to putatively chemically-formed laminations, hence providing for laminations some criteria of biogenicity to be searched for in the fossil record.

## Materials and methods

### Sample collection and fixation

**Figure 1:**
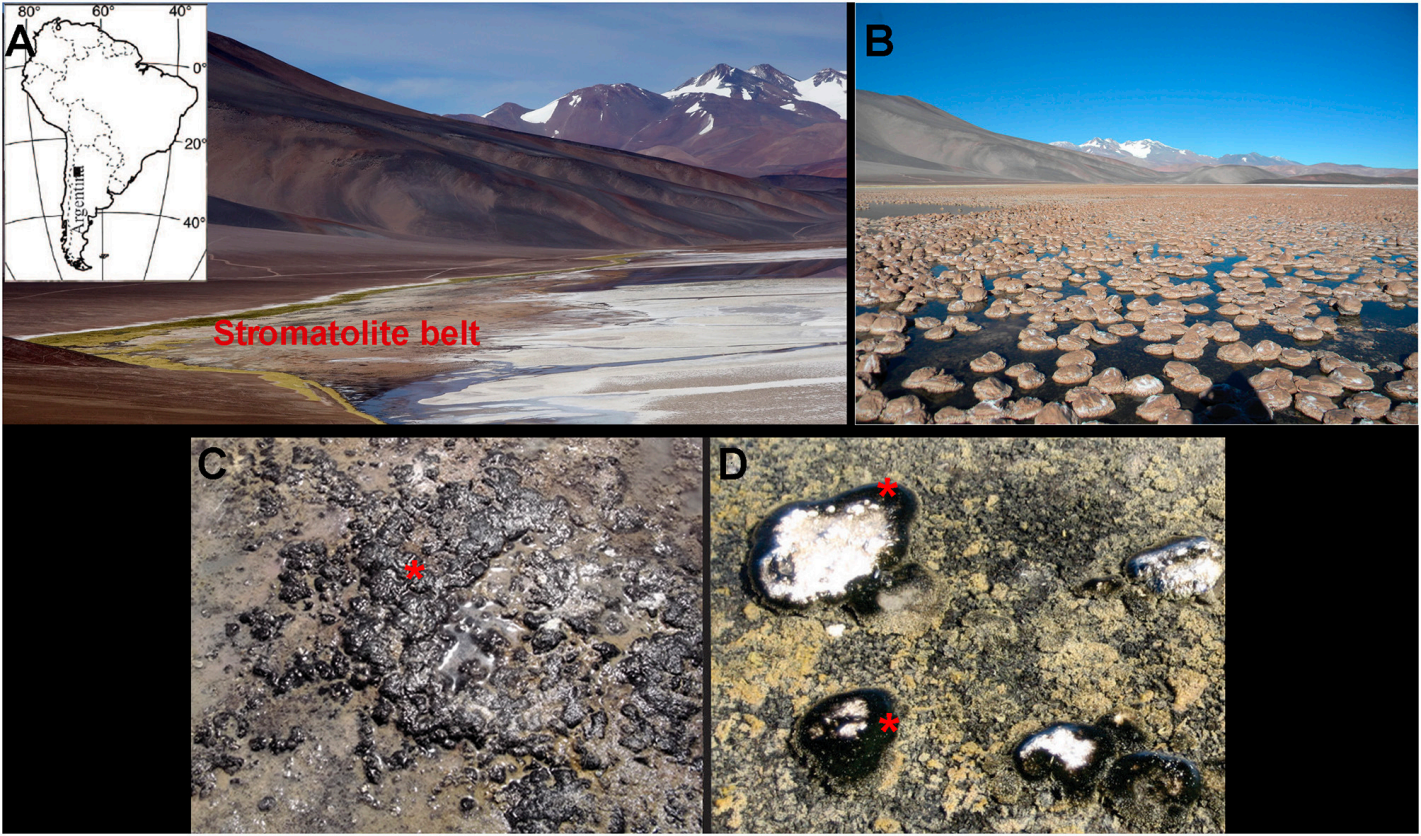
**(A)** Location map of the Laguna Negra (LN) lake in the Catamarca province, Argentina (indicated by a black square) along with a panoramic view of the lake shore and the stromatolite belt. **(B)** Close view of the subspherical oncoids that compose the stromatolite belt. **(C)** Emerged black pustular (BP) mat and **(D)** BP mat covering the periphery of partly emerged oncoids (red asterisks).

The Laguna Negra is a high altitude lake (4,100 m above sea level) located in the Puna region of the Catamarca Province, Argentina (Figure 1A). At the southeastern edge of the Laguna Negra, a stromatolite belt of around 0.3 km^2^ is observed. This belt mostly consists of oncoids and associated microbial mats with morphological and textural differences described in Gomez et al. (2014) (Figures 1B–D). Samples analyzed in this study were collected during two field trips in 2013 (autumn) and in 2015 (spring). We focused on particular black pustular mats usually found in the shallow shore of the lake (Figure 1C), and sometimes covering the periphery of partly emerged oncoids (Figure 1D) where the samples were taken. Mats were sampled with sterile instruments and gloves. Samples for DNA analyses were stored in the dark at −20°C until used, or in RNA*later*® (Ambion, Inc.). Samples for SEM-EDXS were fixed in the laboratory with a 2% glutaraldehyde solution and stored in the dark at 4°C for 2 h. After fixation, samples were washed and progressively dehydrated in a gradual series of ethanol and water baths at increasing ethanol concentrations (i.e., 10, 30, 50, 70, and 100%), prior to air drying or critical point drying (CPD7501, Quorum Technologies). Samples for CLSM were fixed in 4% paraformaldehyde solution directly in the field at 4°C, then washed in phosphate buffered saline (PBS) once back in the laboratory and stored in (1/1) ethanol/PBS solution at −20°C until use. Oncoids and microbialites were also collected and stored at 4°C without any chemical fixation.

### Calcein staining and resin embedding of BP microbialites

Calcein (2,4-bis-[N,N′-di(carbomethyl)-aminomethyl]-fluorescein) produces a stable fluorescent complex in the presence of calcium, strontium, barium, and magnesium ions (Diehl and Ellingboe, 1956) and fluoresces (in the green region of visible light) in the presence of these cations at high pH. These stable fluorescent complexes are integrated in growing carbonates and have been used to stain the growth front of calcium carbonate surfaces in tufa-associated biofilms (Zippel and Neu, 2011) and in living microbialites from Alchichica, Mexico (Gérard et al., 2013). Microbialites samples from the BP mats were stained with calcein (0.1 mg/mL; Merck) at 4°C during 48 h. After staining, microbialite fragments were dehydrated in a gradual series of ethanol baths (30, 50, 70, 90, and 100%), and progressively impregnated with hard grade LR-white resin (Polysciences, Inc.). After polymerization, transverse sections were cut with a diamond wire and polished (diamond powder 0.24 μm) to a final thickness of about 500 μm.

### Confocal laser scanning microscopy (CLSM)

Fresh BP microbial mat samples were stained with Syto®9 (10 μg/mL; ThermoFisher Scientific), a green fluorescent nucleic acid dye. Syto®9-stained samples and resin-embedded microbial mat samples were examined at the Institut de Physique du Globe de Paris (IPGP, Paris, France) using a FluoView™ FV1000 confocal laser scanning microscope with a spectral resolution of 2 nm and a spatial resolution of 0.2 μm (Olympus). The microscope was equipped with a 405 nm laser diode, and multi-line argon (458, 488, and 515 nm), helium-neon-green (543 nm) and helium-neon-red (633 nm) lasers. Fluorescence images were obtained with concomitant excitation at wavelengths of 405, 488, and 543 nm by collecting the emitted fluorescence between 425 and 475 nm, 500 and 530 nm, and 560 and 660 nm, respectively. For CSLM image acquisitions on resin-embedded sections, a water immersion LUMPLFL 60XW objective (Olympus; × 60 magnification) with a numerical aperture (N.A.) of 0.9 was used. For fresh biofilms examination, an oil immersion objective UPLSAPO 60XO (Olympus; × 60 magnification, N.A. = 1.35) was used. 3D images were acquired, visualized, and processed using the F10-ASW FLUOVIEW software (Olympus).

### Bulk DNA extraction, PCR amplification, cloning, and sanger sequencing of 16S rRNA genes

DNA extractions were performed using the MoBio PowerSoil® DNA kit (MoBio) following the instructions provided by the manufacturer. Bacterial 16S rRNA genes were amplified by polymerase chain reaction (PCR) using the bacterial specific primer 27F (5′-AGAGTTTGATCCTGGCTCAG-3′) combined with the universal prokaryotic reverse primer 1492R (5′-GGTTACCTTGTTACGACTT-3′). Archaeal 16S rRNA genes were amplified using the archaeal specific primer 21F (5′-TTCCGGTTGATCCTGCCGGA-3′) and the prokaryote specific reverse primer 1492R. Cyanobacteria 16S rRNA genes were amplified using the cyanobacterial specific primers Cya-106F (5′-CGGACGGGTGAGTAACGCGTGA-3′) and the cyanobacterial specific reverse primer Cya-1387R (5′-TAACGACTTCGGGCGTGACC-3′). One microliter of the extracted DNA was used in a reaction buffer volume of 25 μL containing dNTPs (10 nmol each), 20 pmol of each primer and 1 U GoTaq polymerase (Promega). PCR reactions were performed under the following conditions: 35 cycles (denaturation at 94°C for 15 s, annealing at 55°C for 30 s, extension at 72°C for 2 min) preceded by 2 min denaturation at 94°C, and followed by 7 min extension at 72°C. Cloning was done using the Topo® TA Cloning® system (ThermoFisher Scientific) following the instructions provided by the manufacturer. After plating, positive clones were screened by PCR amplification of inserts using flanking vector primers and the PCR products were partially sequenced (≈700 bp) by GATC Biotech using flanking vector primer T7 (5′-TAATACGACTCACTATAGGG-3′). At least one representative clone per phylotype or Operational Taxonomic Unit (OTU; group of sequences sharing >97% identity) was fully sequenced for Cyanobacteria using flanking vector primer M13R (5′-CAGGAAACAGCTATGAC-3′) for detailed phylogenetic analysis. The sequences have been deposited at the GenBank database and correspond to the accession numbers MH084957 to MH084967 for cyanobacteria, from MH109330 to 109371 for bacteria, and from MH127632 to MH127645 for archaea.

### Laser microdissection, whole genome amplification (WGA), PCR amplification, cloning and sanger sequencing of 16S rRNA genes

Filamentous cyanobacterial cells were isolated using a Zeiss PALM MicroBeam apparatus (Carl Zeiss NTS GmbH) installed in a sterile room at IPGP. We then used the REPLI-g Single Cell Kit (Qiagen) to amplify whole genomic DNA of the microdissected cells. Bacterial 16S rRNA encoding genes were then amplified by PCR using the bacterial specific primer 27F (5′-AGAGTTTGATCCTGGCTCAG-3′) combined with the prokaryote specific reverse primer 1492R (5′-GGTTACCTTGTTACGACTT-3′). One microliter of 1/10 diluted amplified genomic DNA was used in a reaction buffer volume of 25 μL containing dNTPs (10 nmol each), 20 pmol of each primer and 1U of GoTaq polymerase (Promega). PCR reactions were performed under the following conditions: 35 cycles (denaturation at 94°C for 15 s, annealing at 55°C for 30 s, extension at 72°C for 2 min) preceded by 2 min denaturation at 94°C, and followed by 7 min extension at 72°C. Cloning and Sanger sequencing were done as previously described for the 16S rRNA encoding genes retrieved from the bulk DNA extraction. The corresponding sequences have been deposited at the GenBank database and accession numbers range from MH119800 to MH119817.

### PCR amplification, cloning, and sanger sequencing of partial *pufL* and *pufM* genes

Partial *pufL* and *pufM* genes coding for subunits of the photosynthetic reaction center of aerobic anoxygenic photosynthetic bacteria (AAnPB; Koblizek, 2015) were amplified by nested PCR. The first amplification was done using the *pufLM-F* primer (5′-CTKTTCGACTTCTGGGTSGG-3′) and the *pufLM-R* primer (5′-CCCATSGTCCAGCGCCAGAA-3′) (Oz et al., 2005). One microliter of the DNA extracted from the bulk sample was used in a reaction buffer volume of 25 μL containing dNTPs (10 nmol each), 20 pmol of each primer and 1 U GoTaq polymerase (Promega). PCR reactions were performed under the following conditions: 40 cycles (denaturation at 94°C for 15 s, annealing at 50°C for 30 s, extension at 72°C for 2 min) preceded by 2 min denaturation at 94°C, and followed by 7 min extension at 72°C. One microliter of the first amplification products was then used for the nested amplification with the primers Mf150f (5′-AGATYGGYCCGATCTAYCT-3′) and M572r (5′-CCAGTCSAGGTGCGGGAA-3′) (Hirose et al., 2012). The PCR conditions of the first amplification were also used for the nested PCR. As previously described for the 16S rRNA encoding genes, the *pufLM* genes were cloned using the Topo® TA Cloning® system (ThermoFisher Scientific) following the instructions provided by the manufacturer and the PCR products were totally sequenced by GATC Biotech using flanking vector primer T7 (5′-TAATACGACTCACTATAGGG-3′) and M13R (5′-CAGGAAACAGCTATGAC-3′). The corresponding sequences have been deposited in the GenBank database and accession numbers range from MH101761 to MH101777.

### Phylogenetic analysis

Taxonomic affiliations were obtained using BLAST (Altschul et al., 1997) on the non-redundant NCBI database, as well as using the Silva Incremental Aligner (SINA) software (Pruesse et al., 2012). For Cyanobacteria, phylogenetic trees were built with the ARB software (Ludwig et al., 2004) and the SILVA 123 database (Quast et al., 2013; Yilmaz et al., 2014). Representative clones of the dominant phyla were aligned with SINA and then added in the ARB guide tree using the ARB parsimony tool. In addition, the closest environmental 16S rRNA gene sequences retrieved by BLAST were added if they were not present in the SILVA 123 database. Phylogenetic tree was reconstructed using the method of Randomized Accelerated Maximum Likelihood (RAxML) (Stamatakis et al., 2008) with the GTRCAT substitution model. Bootstrap values were calculated from 1,000 replicates.

The phylogenetic tree of partial *pufLM* operons was built using MEGA7 (Tamura et al., 2013), and the Maximum Likelihood method based on the Jukes-Cantor model (Jukes and Cantor, 1969). Initial tree(s) for the heuristic search were obtained automatically by applying Neighbor-Join and BioNJ algorithms to a matrix of pair wise distances estimated using the Maximum Composite Likelihood approach, and then by selecting the topology with the highest log likelihood value. The tree was drawn to scale, with branch lengths measured in the number of substitutions per site. The analysis involved 54 nucleotide sequences. Codon positions included were 1st+2nd+3rd+Noncoding. All positions containing gaps and missing data were eliminated. There were a total of 273 positions in the final dataset.

### Scanning electron microscopy (SEM)

SEM analyses were performed on gold-coated air-dried samples using a Field Emission Zeiss Sigma Scanning Electron Microscope (Carl Zeiss NTS GmbH) at the X Ray Analysis Laboratory (LAMARX, Universidad Nacional de Córdoba, Argentina). SEM observation on carbon-coated samples dried at the critical point were also performed at the Service Commun de Microscopie Electronique à Balayage (UPMC, Paris, France) using a Zeiss Supra 55VP (Carl Zeiss NTS GmbH) SEM equipped with an EDXS spectrometer (X flash Quad detector, Brucker). Images were collected using secondary electron detectors (Everhart-Thornley for high voltage mode, VPSE for variable pressure mode and InLens for low voltage mode) and a backscattered electron detector (AsB). Accelerating voltage ranged from 3 to 15 kV at variable pressures and high current (up to 1 nA).

### Mineralogical identification

Powder X-ray diffraction (XRD) analyses were conducted on BP microbial mat and lithified BP samples using a Philips PW1800/10 powder diffractometer equipped with a Cu anode and a graphite monochromator (LAMARX). Measurements were performed at 40 kV voltage and 30 mA current, from 10 to 60° (2 θ), with an acquisition time of 2.0 s and an angular step of 0.100° 2θ. Resulting diffractograms were analyzed using Highscore software.

### Focused ion beam milling (FIB) and transmission electron microscopy (TEM)

Eight ultrathin electron-transparent sections (~100 nm in thickness) were prepared by FIB milling on an Auriga® FIB-SEM (Carl Zeiss NTS GmbH) available at IPGP using the FIB “lift out” technique (see Heaney et al., 2001 for details). A 30 kV Ga^+^ beam operated at 20 nA was used for the initial steps of the milling. Progressive excavation from both sides of the section area was performed through repeated milling steps. Depth of milling was approximately 7 microns. The final thinning of the section was performed with a less intense Ga^+^ beam operated at 100 pA current.

Transmission Electron Microscope (TEM) observations were carried out on all FIB sections using a JEOL 2100F microscope (JEOL Ltd.) operating at 200 kV at the Institut de Minéralogie, de Physique des Matériaux et de Cosmochimie (IMPMC, Paris, France). The TEM is equipped with a field emission gun, a ultrahigh resolution UHR pole piece and a Gatan energy filter. High-angle annular dark-field scanning transmission electron microscopy (HAADF-STEM) was used with a focused electron beam of a few nm for Z-contrast imaging. Energy Dispersive X-ray Spectrometry (EDXS) analyses were performed using a JEOL detector equipped with an ultrathin window that allowed detection of low Z elements.

### Fourier transform infrared microspectroscopy (FTIR)

Cross-sections of oncoids associated with the black pustular mats were obtained using a sterilized diamond saw and sterile deionized water. They were prepared as doubly-polished sections of about 100 μm thick and without any resin or glue in order to avoid organic contamination. FTIR hyperspectral images were acquired at IPGP using a Thermo Scientific iN10 MX microscope (Ever-Glo™ conventional Infrared source) with a liquid nitrogen cooled detector. Maps were collected in Attenuated Total Reflection (ATR) mode with a germanium tip. Punctual analyses were performed in the 4,000–700 cm^−1^ range, with a spot size of 20 × 20 μm, a spectral resolution of 8 cm^−1^ and with 64 accumulations per spectrum. Background spectra were acquired between each spectrum under the same analytical conditions. Data were processed using the OMNIC™ software (Thermo Fisher Scientific).

### Synchrotron based deep UV fluorescence microspectroscopy (S-DUV)

Slices from oncoid samples associated with black pustular mats were also investigated using synchrotron-based deep UV fluorescence microspectroscopy (S-DUV) and full-field imaging at the French national synchrotron radiation facility SOLEIL (Saint-Aubin, France) on the DISCO beamline (Giuliani et al., 2009). We focused on *Rivularia*-rich laminations in order to better describe the nature and spatial relationships between mineral phases and organic remnants at the micrometer scale. Both TELEMOS and POLYPHEME end stations available on the beamline were used. Samples were first investigated by full-field luminescence microscopy (TELEMOS end station) using an Axio ObserverZ1 microscope (Carl Zeiss MicroImaging) with a × 40 objective. The excitation wavelength was set at 275 nm with a DM300 dichroic mirror. Fluorescence emission was collected using 3 filters (bandpass at 327–353 nm, 370–410 nm, and 420–480 nm). Acquisition time was set at 30 s for all channels except for the 327–353 nm filter (60 s) and 2D fluorescence images were acquired along a transect, perpendicularly to the laminations of the oncoid sample. Images obtained with TELEMOS microscope were treated with ImageJ software (Schneider et al., 2012) and allowed localizing areas with the highest fluorescent signals. We then selected specific areas with *Rivularia*-like morphologies on which hyperspectral fluorescence maps were acquired (POLYPHEME end station) on an Olympus IX71 inverted microscope with homemade replacement of the intermediate lenses set to be transparent in the deep UV range (Jamme et al., 2010, 2013; Thoury et al., 2011) and a × 40 objective. Excitation was set at 275 nm and a bypass mode was used in order to increase the intensity of the collected fluorescence signal. The collection range was set from 310 to 600 nm. Spectra were processed using LabSpec software (Horiba Scientific).

## Results

### Description of the microbial mat and associated oncoids

**Figure 2:**
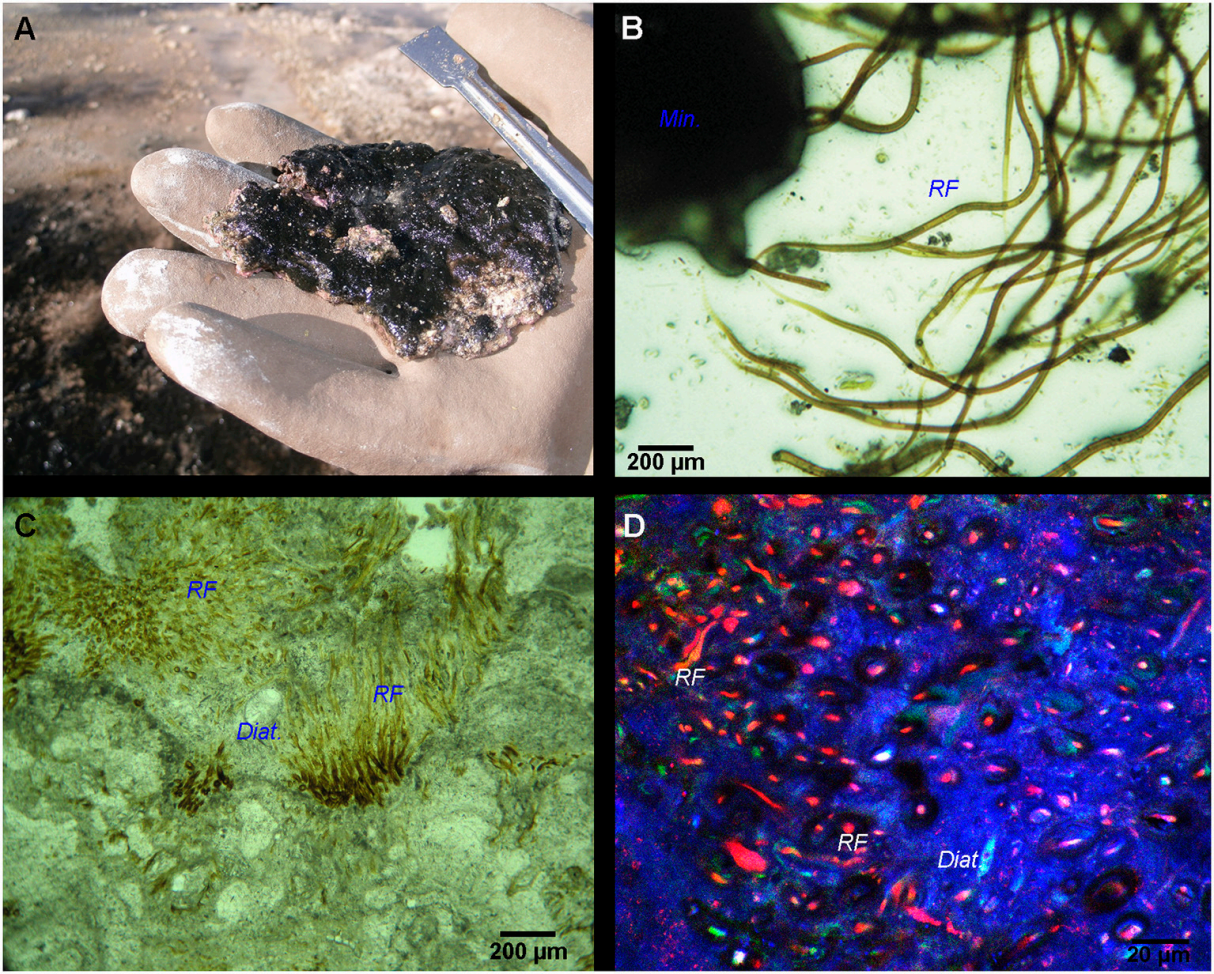
**(A)** Black pustular mat with visible white-colored carbonate precipitates. **(B)** Optical microscopy image of the BP mat highlighting *Rivularia* filaments (*RF*) associated with minerals (*Min*.). **(C)** Paintbrush-like palisade fabric of *Rivularia*-like filaments encrusted by carbonates and preserved inside an oncoid lamination alternating with other lamination types (like sparry, botryoidal and micritic). Encrusted diatoms (*Diat*.) can also be found. **(D)** CLSM image of the same lamination enclosing *Rivularia*-like filaments whose remaining pigments show red fluorescence. The mineralized portion of the oncoid appears in blue due to laser reflection on solid carbonates.

The microbial mat was characterized by a bulbous to pustular morphology (Figure 1C) and measured 1 to 8 mm in thickness. It had a dark pigmentation (Figures 1C,D), possibly due to the abundancy of scytonemin, a photoprotective pigment produced under high UV irradiation (Garcial-Pichel and Castenholz, 1991). No clear lamination was visible beneath the surface layer, although mixed green, pink and black patches along with white carbonated patches were detected (Figure 2A). Optical observation of this mat (Figure 2B) highlighted the predominance of large filamentous microorganisms resembling cyanobacterial *Rivularia* sp. (as confirmed by Sanger sequencing) and measuring around 12 to 15 μm in diameter.

Irregular laminations with dark brown to yellowish vertically oriented *Rivularia* filaments (15–20 μm in diameter) were observed in petrographic sections of associated oncoids (Figure 2C). They alternated with micritic or botryoidal laminations. They presented a tufted paintbrush-like palisade fabric (Reitner et al., 1996). CLSM observations of these petrographic sections carried out at an excitation of 633 nm revealed a strong fluorescence of the *Rivularia* filaments within the paintbrush-like laminations (in red in Figure 2D). Some pigments were also observed near the filament sheaths using excitation at 543 nm.

### Microbial associations in BP mats

**Figure 3:**
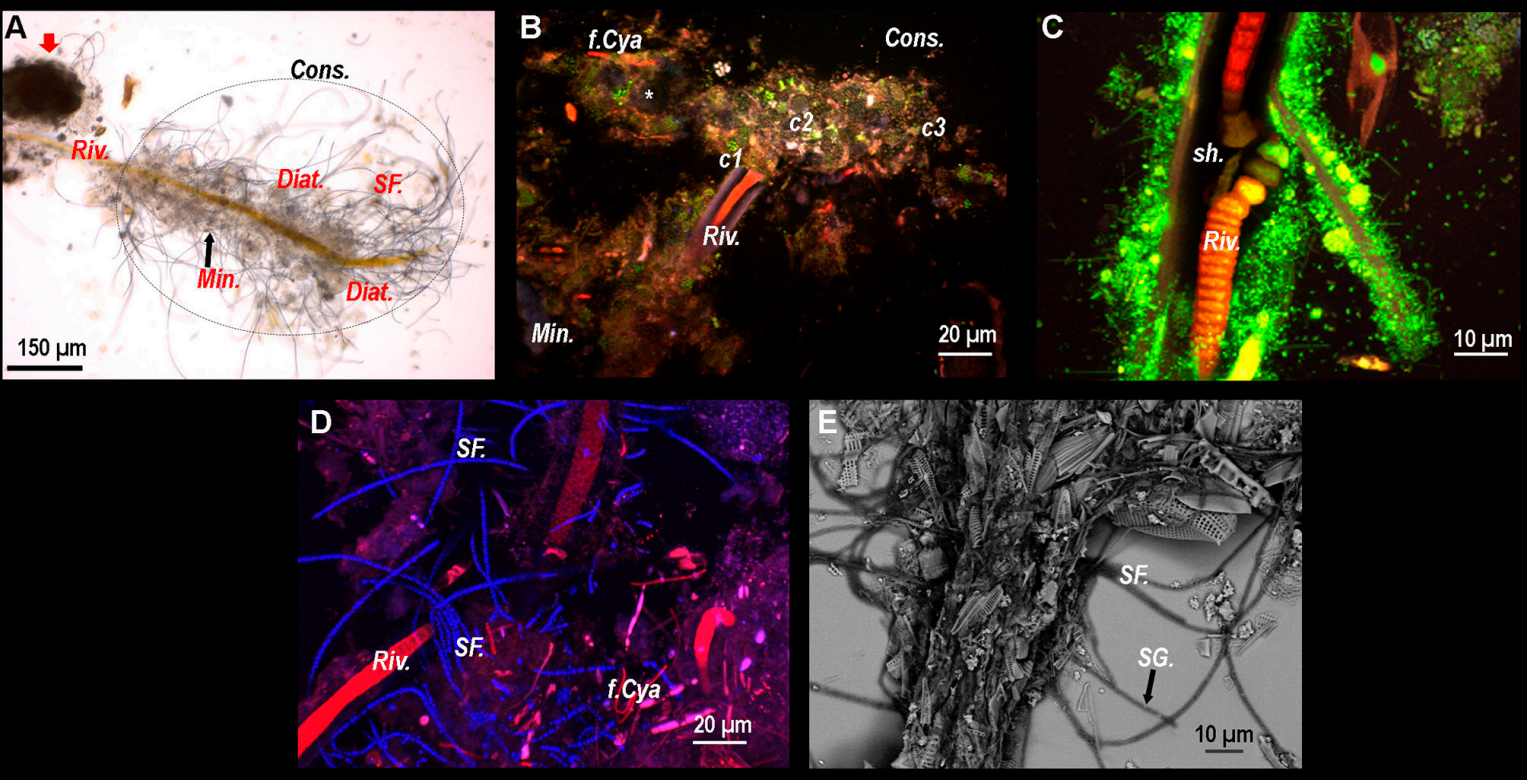
**(A)** Optical microscopy image of a *Rivularia* filament (*Riv*.) with which numerous microorganisms are associated, hence suggesting the presence of a consortium (*Cons*.) (dotted circle). A large mineral aggregate is also visible near the filament (red arrow). Sulfur filaments (*SF*.), diatoms (*Diat*.) and mineral grains (*Min*.) can also be observed around the filament. From **(B–D)**: Composite CSLM images obtained over an integrated depth of 50 μm by concomitant excitations at 405, 488, and 543 nm and collection between 425 and 475 nm, 500 and 530 nm, and 560 and 660 nm, respectively. **(B)** Composite CLSM image of a consortium surrounding a *Rivularia* filament distinguished in red due to its photosynthetic pigments. Inside the consortium, abundant cocci-shaped cells are observed thanks to Syto®9 staining (green) (*c1, c2*, and *c3*). Minerals appear in light blue due to laser reflection (white asterisk). Other filamentous cyanobacteria (*f.Cya*) are observed. **(C)** High magnification composite CLSM image of a *Rivularia* filament where cells are visible (in red) inside the sheath (*sh*.), which is covered by a myriad of Syto®9-stained cells. **(D)** Detail of a *Rivularia* filament associated with sulfur-bearing bacterial filaments (bright blue dots due to laser reflection). **(E)** Magnified SEM image in backscattered electron mode showing a *Rivularia* filament and the associated sulfur-bearing filaments (black arrow) with sulfur grains (*SG*.) appearing as bright dots. **(A,B,D,E)** were obtained on BP13 mat samples, while **(C)** was observed on BP15 mat sample after laser microdissection.

Conventional optical microscopy revealed the presence of a strongly mineralized microbial consortium associated with the *Rivularia*-like filaments. This latter included sulfur-bearing bacterial filaments and diatoms (Figure 3A). This consortium was also observed by CLSM. Filamentous cyanobacteria were identified based on the auto-fluorescence of their photosynthetic pigments (in red in Figure 3B). Numerous cocci (*c1, c2*, and *c3*) and filamentous cells were also observed, fluorescing in green after Syto®9 staining. Among them, colonies of cells with a peculiar coccus shape (*c1*) were detected in close association with the *Rivularia* sheaths and the minerals (in light blue in Figure 3B). At high magnification, abundant microorganisms were observed in association with the thick sheath of a *Rivularia* filament (in green in Figure 3C). *Rivularia* cells were also visible (in red in Figure 3C). Consistently with conventional optical microscopy, filamentous sulfur-bearing bacteria were also detected in bright blue (laser reflection on the sulfur grains, Figure 3D) close to the *Rivularia* filaments. This was also confirmed by SEM imaging highlighting elemental sulfur grains on these filaments (*SF*. in Figure 3E). No precipitated mineral was detected in close relationship with the *Rivularia* sheaths.

### Microbial diversity

In order to characterize the microorganisms forming the consortium associated with the *Rivularia* filaments and to assess their potential role in mineral precipitation, 16S rRNA gene sequences of Bacteria and Archaea were analyzed for the BP mat samples collected in 2013 and 2015. Results obtained from either laser microdissected cyanobacterial filaments collected in 2015 or bulk DNA extractions on all samples are summarized in the following sections.

#### Laser microdissection and whole genome amplification of *Rivularia* filaments and of the associated bacterial consortium

After laser microdissection of large filamentous cyanobacteria, we detected three different representative 16S rRNA gene sequences (3c-25, 3c-38, and 3c-57) of cyanobacteria affiliated to the *Rivularia* genus (Table 1). 16 sequences represented by 3c-25 and 3c-57 shared 99% identities with the 16S rRNA gene sequence of *Rivularia* detected in a microbial mat associated with the microbialites of Alchichica Lake (JN825310) and *Rivularia atra* BIR MGR1 (AM230675), a marine species forming black colonies (Guiry and Morrison, 2015) and *Calothrix* sp. XP9A (AM230670). 14 sequences represented by 3c-38 share 100% identities with *Rivularia* sp. PUNA_NP3_PCI185B (KY296608) isolated from Laguna Negra microbial mat. Heterotrophic Bacteroidetes affiliated to *Marivirga* (Pagani et al., 2011), *Maribacter* and *Winogradskyella* genera constituted the most abundant epiphytic bacterial community associated with the *Rivularia* filaments (Table 1). No archaeal sequences were detected within the consortium, hence suggesting that archaea were not closely associated with the *Rivularia* filaments.

**Table 1:** Taxonomic affiliations of the bacterial 16S rRNA gene sequences retrieved using general bacterial primers after laser microdissection from the BP consortia collected in 2015.

	**Taxonomy**	**Nb**	**Closest environmental bacteria**	**Closest cultivated bacteria**
4c-1	Bacteroidetes; Flammeovirgaceae; *Marivirga*	4		KT324862 100%, *Marivirga* sp. CR-23
4c-14	Bacteroidetes; Flammeovirgaceae; *Marivirga*	5	AF170787 98%, Antarctic quartz stone sublithic communities	NR112183 98%, *Marivirga sericea* strain IFO 15983
3c-4	Bacteroidetes; Flavobacteriaceae	3	AF170787 96%, phycosphere of *Enteromorpha prolifera*	JQ069961 96% *Maribacter* sp. BSw21901
3c-13	Bacteroidetes; Flavobacteriaceae; *Maribacter*	3		JQ988061 99%, *Maribacter* sp. T28
3c-34	Bacteroidetes; Flavobacteriaceae	2		NR_043453 99%, *Psychroserpens mesophilus*
3s-2	Bacteroidetes; Flavobacteriaceae	8		JQ 687107 99%, *Winogradskyella* sp. KYW 630
3s-13	Bacteroidetes; Flavobacteriaceae; *Winogradskyella*	4		NR_137338 95%, *Winogradskyella litoriviva*
3c-11	Bacteroidetes; Flavobacteriaceae; *Winogradskyella*	1	KY190901 95%, marine sediment, Antartica	AY771731 95%, *Winogradskyella thalassocola*
3c-25	Cyanobacteria; SubsectionIV; FamilyII; *Rivularia*	14	JN825310 99%, microbialites from Alchichica alkaline lake	AM230675 99%, *Rivularia atra* BIR MGR1
3c-38	Cyanobacteria; SubsectionIV; FamilyII; *Rivularia*	14		KY296608 100%, *Rivularia* sp. PUNA_NP3_PCI185B
3c-57	Cyanobacteria; SubsectionIV; FamilyII; *Rivularia*	2	JN825310 99%, microbialites from Alchichica alkaline Lake	AM230670 99%, *Calothrix* sp. XP9A
3c-51	Cyanobacteria; SubsectionI; FamilyI; *Gloeocapsa*	3	GQ340127 98%, water column Marathonas Reservoir	GQ375048 98%, *Limnococcus limneticus*
3c-43	Proteobacteria; Gammaproteobacteria; HTA4	2	JQ586297 98%, arctic marine sediment	EF492067 97%, Candidatus *Berkiella cookevillensis*
3s-16	Proteobacteria; Gammaproteobacteria; Legionellaceae; *Legionella*	2		LT906452 96%, *Legionella pneumophila*
4c-9	Unclassified	6	AB630669 96%, aquatic moss pillars	
3c-15	Unclassified	3	KJ998102 97%, Guerrero Negro intertidal mat	
3c-45	Unclassified	1	KJ998102 95%, Guerrero Negro intertidal mat	

*Nb stands for number of clones*.

#### Bulk DNA extraction

**Figure 4:**
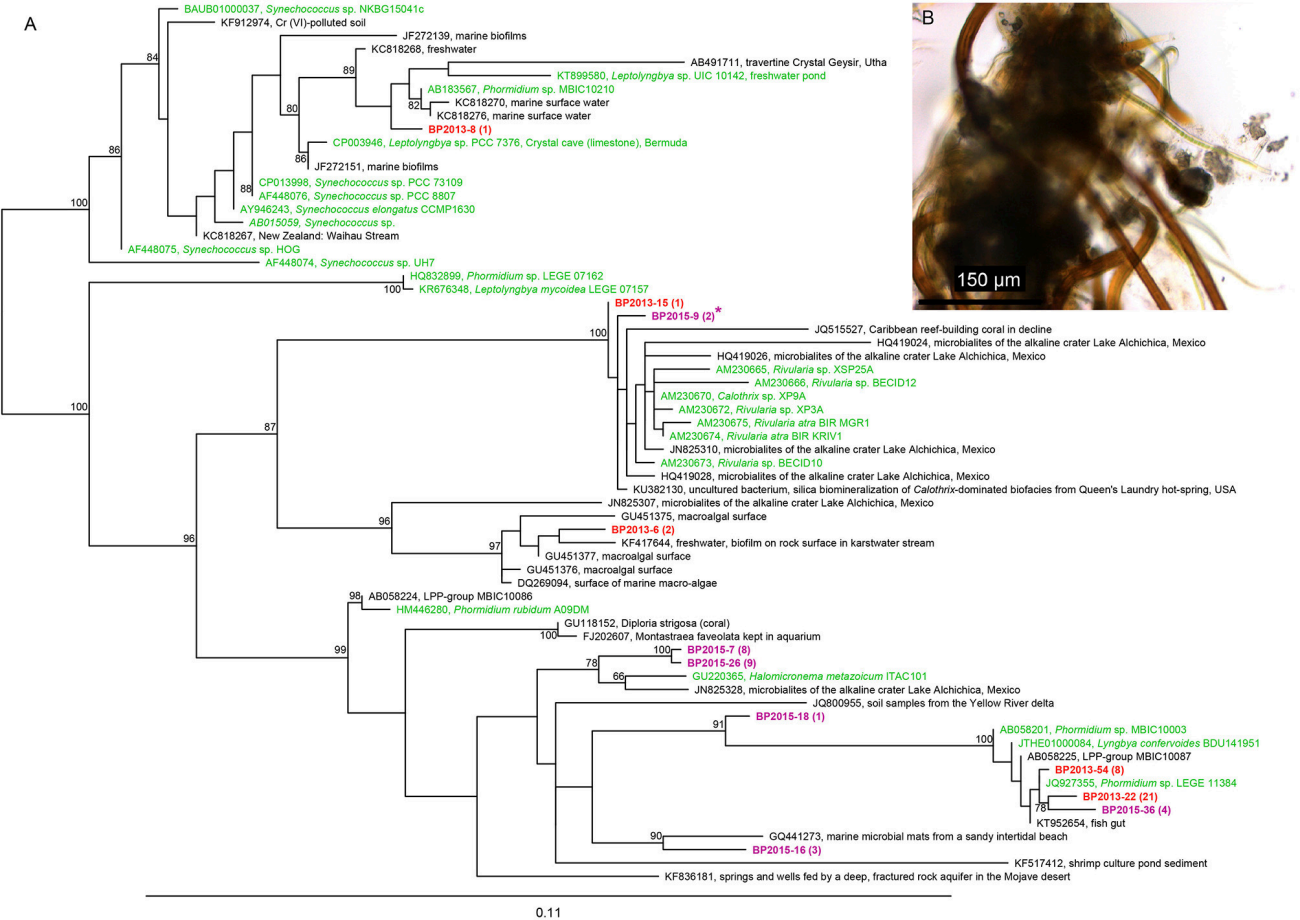
**(A)** Phylogenic tree of the 16S rRNA encoding gene sequences of cyanobacteria retrieved using the cyanobacterial specific primers in the black pustular mats in 2013 (red) or 2015 (purple). Numbers in parentheses represent the number of clones obtained for each operational taxonomic unit (OTU) defined at a sequence similarity ≥97%. The cyanobacteria sequence identified in the consortium isolated by laser microdissection corresponds to BP2015-9^*^. The tree was constructed with the ARB software (Ludwig et al., 2004) by Maximum Likelihood analysis using 1,097 positions, by including the closest possible uncultivated (black) and cultivated (green) relatives as well as more distant representatives of cultivated species. Bootstrap values for nodes (>70% support) based on 1,000 replicates are displayed as percentages. **(B)** Optical microscopy image of one of the consortia isolated by laser microdissection and showing *Rivularia* filaments.

After bulk DNA extraction, cyanobacteria affiliated to the *Rivularia* genus were detected in both mats collected in 2013 and 2015 using cyanobacterial specific primers (Figure 4) but not using universal bacterial primers. The 16S rRNA gene sequences of *Rivularia* species detected in 2015 (BP2015-9^*^) were identical to the 3c-25 sequences detected after whole genome amplification (Figure 4). Yet, the most abundant cyanobacterial 16S rRNA gene sequences detected using cyanobacterial specific primers were affiliated to the *Phormidium* genus for both 2013 and 2015 (Figure 4). The clones were notably affiliated to *Phormidium* sp. MBIC10210 LEGE 11384 and *Phormidium lucidum* CY-012 (JQ927355 and KC217548, respectively; 98% to 99% identities). 17 sequences from 2015 were also closely related to sequences detected in microbialites from Alchichica, an alkaline Mexican lake (*Halomicronema* sp., JN825328; 97% identity).

A description of the main bacterial sequences detected in 2013 and 2015 using general bacterial primers is given in Table 2. Bacteroidetes, Proteobacteria and Verrucomicrobia were the most abundantly detected phyla. Only one sequence of cyanobacteria was detected. Among the bacterial phyla, Bacteroidetes belonging to the *Lewinella* genus were identified. Their cultivable representatives (*Lewinella cohaerens, Lewinella persica*) correspond to orange to black chemoorganotrophic bacteria (Khan et al., 2007). Interestingly, members of the Gammaproteobacteria found in both 2013 and 2015, samples have closest uncultivated bacteria (98% identities) that were only detected in the Altiplano at Salar de Ascotan in Chile (EF632661). Bacteria belonging to the Myxococcales order were also observed in both samples. They were affiliated to bacteria from the semiarid “Tablas de Dainiel National Park” wetland (Central Spain, FJ516764).

**Table 2:** Taxonomic affiliations of the bacterial 16S rRNA gene sequences using general bacterial primers retrieved from the BP mats collected in 2013 (gray) and 2015 (white).

	**Taxonomic affiliation**	**Nb**	**Closest uncultured bacterium**	**Closest cultivated bacterium**
BPb2015-4	Bacteroidetes; Cryomorphaceae;	1	JQ197952 98%, sea water	CP003156 89%, *Owenweeksia hongkongensis*
BPb2015-3	Bacteroidetes; Flavoceae; *Psychroflexus*	3	AY298788 98%, diatom detritus Southern Ocean sea water	EU000243 97%, *Donghaeana dokdonensis*
BPb2015-15	Bacteroidetes; Flavoceae; *Psychroflexus*	1	GU437550 94%, sediment El Tatio Geyser Field, Chile	
BPb2015-2	Bacteroidetes; Flavoceae; *Psychroflexus*	1	JN453999 95%, Guerrero Negro hypersaline microbial mat	
BPb2015-27	Bacteroidetes; Flavoceae*; Psychroflexus*	1	GU437622 97%, sediment El Tatio Geyser Field, Chile	NR_108235 96%, *Psychroflexus salinarum*
BPb2015-30	Bacteroidetes; Flavoceae; *Psychroflexus*	1	EF190068 94%, Qinghai oilfield	
BPb2013-6	Bacteroidetes; Flavoceae; *Winogradskyella*	2		JQ661183 97%, *Winogradskyella echinorum*
BPb2013-36	Bacteroidetes; Flavoceae; *Winogradskyella*	3		JQ661183 97%, *Winogradskyella echinorum*
BPb2013-13	Bacteroidetes; Flavobacteraceae	1		KJ475165 94%, *Psychroserpens damuponensis*
BPb2013-19	Bacteroidetes; Flavobacteraceae	1	AY298788 98%, diatom detritus	CP025116 96%, *Nonlabens* sp. MB-3u-79
BPb2013-2	Bacteroidetes; Saprospiraceae; *Lewinella*	1		EU371935 95%, *Lewinella persicus*
BPb2013-32	Bacteroidetes; Saprospiraceae; *Lewinella*	1		JQ661170 95%, *Lewinella agarilytica*
BPb2015-12	Bacteroidetes; Saprospiraceae; *Lewinella*	1	JQ753202 96%, Antarctic sea ice	NR_112672 94%, *Lewinella cohaerens*
BPb2015-21	Bacteroidetes; Saprospiraceae; *Lewinella*	4		KY009734 96%, *Lewinella* sp. SD302
BPb2015-33	Bacteroidetes;	1	FJ213812 94%, Altiplano, Salar de Ascotan, Chile	
BPb2015-14	Cyanobacteria; SubsectionIII; Phormidium	1		JQ927355 98%, *Phormidium* sp. LEGE 11384
BPb2015-26	Proteobacteria	1	LC213232 96%	
BPb2015-17	Proteobacteria; Alphaproteobacteria; Hyphomonadaceae	1	JN530502 96%, Guerrero Negro hypersaline microbial mat	NR_148267 94%, *Hyphomonas beringensis*
BPb2015-1	Proteobacteria; Alphaproteobacteria; Hyphomonadaceae	1	JN436614 99%, Guerrero Negro hypersaline microbial mat	CP017718 98%, *Hyphomonas* sp. Mor2
BPb2015-22	Proteobacteria;Alphaproteobacteria; Parvularculaceae	2	GU326496 97%, desalinisation plant	
BPb2013-3	Proteobacteria; Alphaproteobacteria; Rhodobacteraceae	1	AM990873 97%, Mediterranean Sea	HE962517 95%, *Tropicibacter mediterraneus*
BPb2013-30	Proteobacteria; Alphaproteobacteria; Rhodobacteraceae	1	GQ441231 96%, marine microbial mats sandy beach	KJ486297 93%, *Roseinatronobacter* sp. MOL1.10
BPb2013-5	Proteobacteria; Alphaproteobacteria; Rhodobacteraceae	1	GU083689 96%, Inner Mongolia, Xiarinur soda lake	NR_044285 96%, *Rhodobaca barguzinensis*
BPb2013-8	Proteobacteria; Alphaproteobacteria; Rhodobacteraceae	1	KJ475514 97%, Oil-derived marine aggregates	JX861563 97%, *Tropicibacter* sp. MCCC1A07686 l
BPb2015-18	Proteobacteria; Alphaproteobacteria; Rhodobacteraceae	1		KY770546 98%, *Oceanicola* sp. strain 7002-119
BPb2015-9	Proteobacteria; Alphaproteobacteria; Rhodobacteraceae	1		KJ486297 97%, *Roseinatronobacter* sp. MOL1.10
BPb2013-16	Proteobacteria; Alphaproteobacteria; Rhodobacteraceae; *Marivita*	1	KY770575 98%, phycosphere	NR_044514 98%, *Marivita cryptomonadis*
BPb2015-11	Proteobacteria; Alphaproteobacteria; Rhodobacteraceae; *Octadecabacter*	1		KX073749 98%, *Octadecabacter* sp. HDSW-34
BPb2013-7	Proteobacteria; Alphaproteobacteria; Rhodobacteraceae*; Sulfitobacter*	1		NR_134206 99%, *Sulfitobacter noctilucicola* costal
BPb2015-24	Proteobacteria; Alphaproteobacteria; Rhodobacteraceae	1		KF418804 94%, *Sulfitobacter* sp. S19SW
BPb2015-6	Proteobacteria; Gammaproteobacteria; Alteromonadaceae*; Marinobacter*	1		NR_145917 93%, *Marinobacter confluentis*
**BPb2013-11**	**Proteobacteria; Gammaproteobacteria;**	**1**	**EF632661 98%, Chile: Altiplano, Salar de Ascotan**	
**BPb2015-34**	**Proteobacteria; Gammaproteobacteria;**	**1**	**EF632661 98%, Chile: Altiplano, Salar de Ascotan**	
BPb2015-28		1		
BPb2015-23	Proteobacteria; Gammaproteobacteria; Oceanospirillaceae*;Nitrincola*	1		FJ764761 96%, *Nitrincola* sp. E-044
BPb2015-5	Proteobacteria; Gammaproteobacteria; OM182 clade	1	HM127577 98%, Qinghai Lake	NR_112620 91%, *Thioprofundum hispidum*
BPb2013-28	Proteobacteria; Gammaproteobacteria	1	EF632659 99%, aquatic environment Altiplano Chile	MG264256 98%, *Wenzhouxiangella marina*
**BPb2015-55-CYA**	**Proteobacteria; Deltaproteobacteria; Myxococcales**	7^*^	**FJ516764 97%, wetland (Central Spain)**	
**BPb2013-14-CYA**	**Proteobacteria; Deltaproteobacteria; Myxococcales**	6^*^	**FJ516764 99%, wetland (Central Spain)**	
BPb2013-10	Verrucomicrobia; Verrucomicrobiaceae; *Haloferula*	3	KY190897 95%, marine sediment from Potter Cove	NR_109435 95%, *Haloferula chungangensis*
BPb2015-7	Verrucomicrobia	2	JN480742 96%, Guerrero Negro hypersaline microbial mat	

Two non-cyanobacterial sequences were retrieved with the specific cyanobacterial primers (^*^). For the sequences retrieved using the cyanobacterial specific primers, please refer to Figure 4. Strictly identical sequences retrieved in BP2013 and BP2015 are indicated in bold. Nb stands for number of clones.

The other detected bacterial species were specific to the BP mats collected in 2013 or 2015. In the BP mat collected in 2013, numerous bacteria affiliated to marine species were identified. Bacteroidetes closely related to *Winogradskyella echinorum* (JQ661183, 97% identity; Nedashkovskaya et al., 2009) were detected. Most of the observed Alphaproteobacteria clones belonged to the Rhodobacteraceae family. One OTU was closely related to the *Tropicibacter* genus (97% identity; Lucena et al., 2013) and another one to *Sulfitobacter noctilucicola* (NR_134206, 99% identity; Kwak et al., 2014). An additional member of Alphaproteobacteria was closely related (98% identity) to the aerobic phototrophic marine bacteria *Marivita cryptomonadis* (NR_044514). Finally, Verrucomicrobia members affiliated to the *Haloferula* genus were distinguished. They were close to *Haloferula chungangensis* (NR_109435), a heterotrophic ureolytic bacterium from marine sediments (Kang et al., 2014). Conversely, the sulfur-bearing filamentous cells observed using CLSM and SEM in association with *Rivularia* (Figures 3A,D,E) were not clearly identified in our 16S rRNA gene sequences retrieved by bulk DNA extraction. Although the unidentified sulfur-bearing filament bacteria were morphologically similar to *Thiotrix* species (Howarth et al., 1999), further efforts will be required to identify them by testing new protocol of DNA extraction retrieving additional 16S rRNA encoding gene sequences and performing laser microdissection.

In the BP mat collected in 2015, several detected bacteria were closely related to uncultivated bacteria from the Guerrero Negro hypersaline microbial mats belonging to Bacteroidetes (JN453999), Alphaproteobacteria (JN530502, JN436614) and Verrucomicrobia (JN480742). Other identified Bacteroidetes were close to bacteria from El Tatio hot springs (GU437622), a high altitude (4400 meters above sea level) geothermal site with low sulfide and high arsenic concentrations in Chile (Engel et al., 2013). These Bacteroidetes were also related to *Psychroflexus salinarum* (NR108235; 96% identity), which correspond to dark orange bacteria isolated from marine solar saltern (Yoon et al., 2008). Moreover, three bacterial sequences affiliated to Bacteroidetes already found associated with diatom detritus were also observed in this sample (AY298788; 98% identity). Similarly to the results obtained for the 2013 BP mat, several sequences affiliated to the *Roseobacter* clade were detected and belonged to the *Oceanicola* genus (98% identities, KY770546), the *Octadecabacter* genus (98% identity with KX073749) and the *Roseinatronobacter* genus (97% identities with KJ486297), all these bacteria being aerobic phototrophic bacteria.

As observed for Bacteria, the Archaea communities were clearly different in BP mats collected in 2013 and in 2015 (Table 3). In 2013 BP samples, we detected only methanogenic archaea. Sequences affiliated to the obligate acetoclastic methanogenic *Methanosaeta harundinacea* (Ma et al., 2006) (CP003117, 99% identity). In addition, some sequences were related to *Methanolinea tarda* (NR_028163, 97% identity), a strain using H_2_ and formate for growth and methane production (Imachi et al., 2008). Diverse other methanogenic archaea were detected, notably some closely affiliated to *Methanogenium cariaci* (99% identities) a marine methanogenic archaea (N_104730, 99% identities), which uses hydrogen and carbon dioxide as substrates for growth (Romesser et al., 1979). In 2015 BP, the archaea detected were affiliated to the Thermoplasmatales order. The sequences were related to 16S rRNA gene sequences of uncultivated archaea retrieved in hypersaline mats (EU585947, EU585956, HM480251).

**Table 3:** Taxonomic affiliations of the archaeal 16S rRNA gene sequences using general archaeal primers retrieved from the BP mats collected in 2013 (gray) and 2015 (white). Nb stands for number of clones.

	**Taxonomic affiliation**	**Nb**	**Closest uncultured archaeon**	**Closest cultivated archaeon**
2015BPar-1	unclassified	1	EU585947 95%, hypersaline microbial mat	
2015BPar-21	Euryarchaeota; Thermoplasmatales; Marine Benthic Group D and DHVEG-1	2	EU585947 99%, hypersaline microbial mat	
2015BPar-30	Euryarchaeota; Thermoplasmatales; Marine Benthic Group D and DHVEG-1	2	EU585956 98%, hypersaline microbial mat	
2015BPar-19	Euryarchaeota; Thermoplasmatales; Marine Benthic Group D and DHVEG-1	1	EU585961 96%, hypersaline microbial mat	
2015BPar-16	Euryarchaeota; Thermoplasmatales; Marine Benthic Group D and DHVEG-1	1	EU585964 97%, hypersaline microbial mat	
2015BPar-13	Euryarchaeota; Thermoplasmatales; Marine Benthic Group D and DHVEG-1	1	HM480251 99%, hypersaline microbial mat	
2015BPar-9	Euryarchaeota; Thermoplasmatales; Marine Benthic Group D and DHVEG-1	1	EU585964 98%, hypersaline microbial mat	
2013BPar-30	Euryarchaeota; Methanosarcinales; Methanosaetaceae; Methanosaeta	1	HG001405 99%, salt marsh sediment	CP003117 98%, *Methanosaeta harundinacea*
2013BPar-26	Euryarchaeota; Methanosarcinales; Methanosaetaceae; Methanosaeta	6	KX581173 99%, marine sediment	CP003117 99%, *Methanosaeta harundinacea*
2013BPar-3	Euryarchaeota; Methanosarcinales; Methanosarcinaceae;	2	KP987245 99%, subsurface sediments	KF952458 95%, *Methanosalsum zhilinae*
2013BPar-24	Euryarchaeota; Methanosarcinales; Methanosarcinaceae;	3	KP987245 99%, subsurface sediments	NR_102894 97%, *Methanosalsum zhilinae*
2013BPar-2	Euryarchaeota; Methanomicrobiales; Methanoregulaceae; Methanolinea	2	MG062727 99%, anaerobic granules	NR_112799 96%, *Methanolinea mesophila*
2013BPar-9	Euryarchaeota; Methanomicrobiales; Methanoregulaceae; Methanolinea;	6	AB236052 98%, marine sediment	NR_028163 97%, *Methanolinea tarda*
2013BPar-11	Euryarchaeota; Methanomicrobiales; Methanomicrobiaceae; Methanogenium	2		NR_104730 99%, *Methanogenium cariaci*

#### *pufLM* genes cluster detection

**Figure 5:**
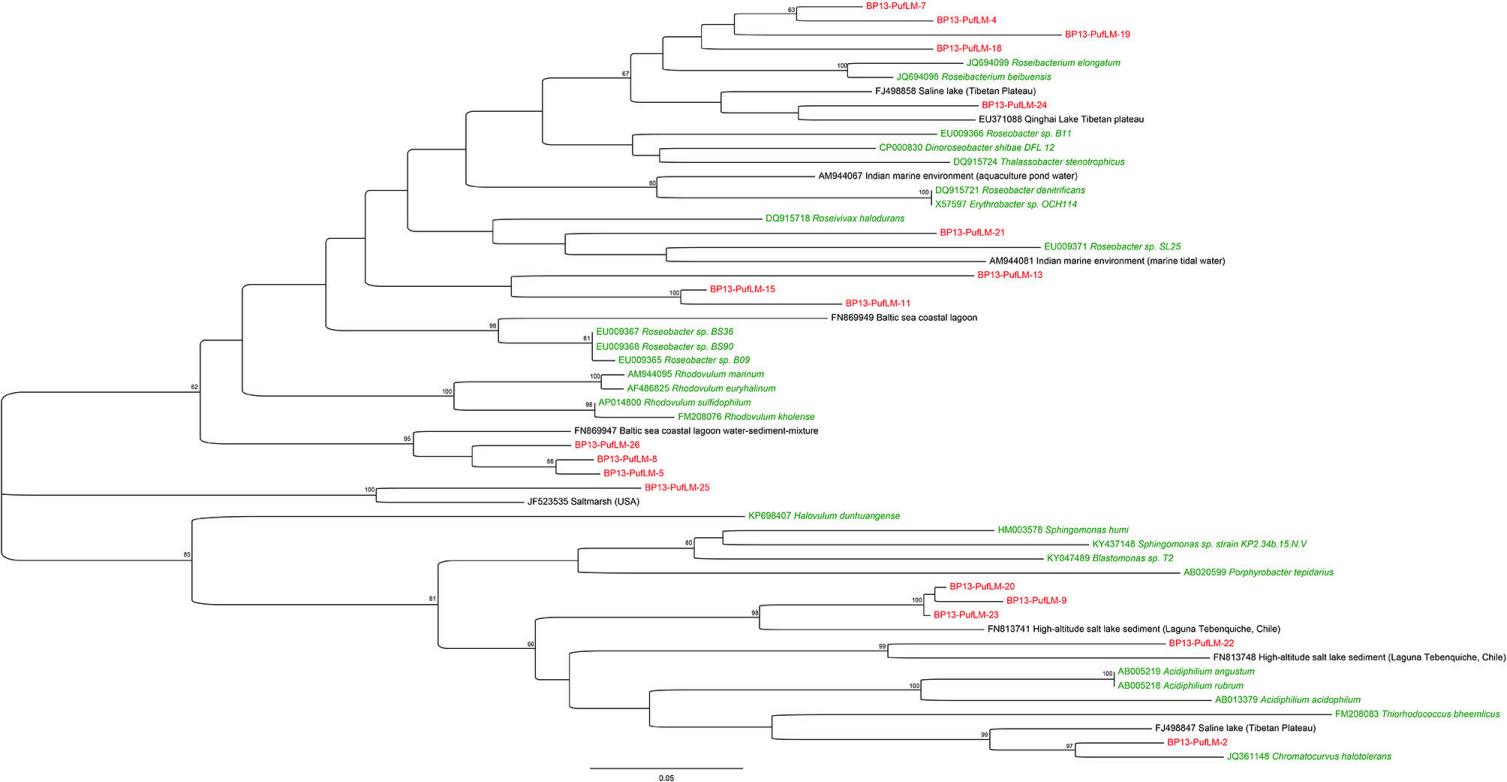
Maximum Likelihood phylogenetic analysis of partial *pufLM* operons detected in the black pustular mat collected in 2013. The evolutionary history of the retrieved partial *pufLM* operons and of the partial *pufLM* operons of the closest uncultivated (black) and cultivated (green) relatives was inferred by using the Jukes-Cantor model (Jukes and Cantor, 1969). The tree with the highest log likelihood (−6611.4021) is shown. The percentage of trees in which the associated taxa clustered together is shown next to the branches. The tree is drawn to scale, with branch lengths measured in the number of substitutions per site. Evolutionary analyses were conducted using MEGA7 (Tamura et al., 2013).

The phylogenetic analysis of the *puflM* genes cluster detected in 2013 (BP13) showed that at least 18 different sequences of aerobic anoxygenic phototrophic bacteria (AAnPB) were present in the analyzed BP mat. As also noticed for the 16S rRNA encoding genes, two groups of AAnPB seemed to be endemic of high altitude Andean lakes as the partial *pufLM* sequences were close to sequences detected in a high altitude salt lake in Chile (FN813741 and FN813748) (Figure 5).

### Associated carbonate minerals in BP mats

**Figure 6:**
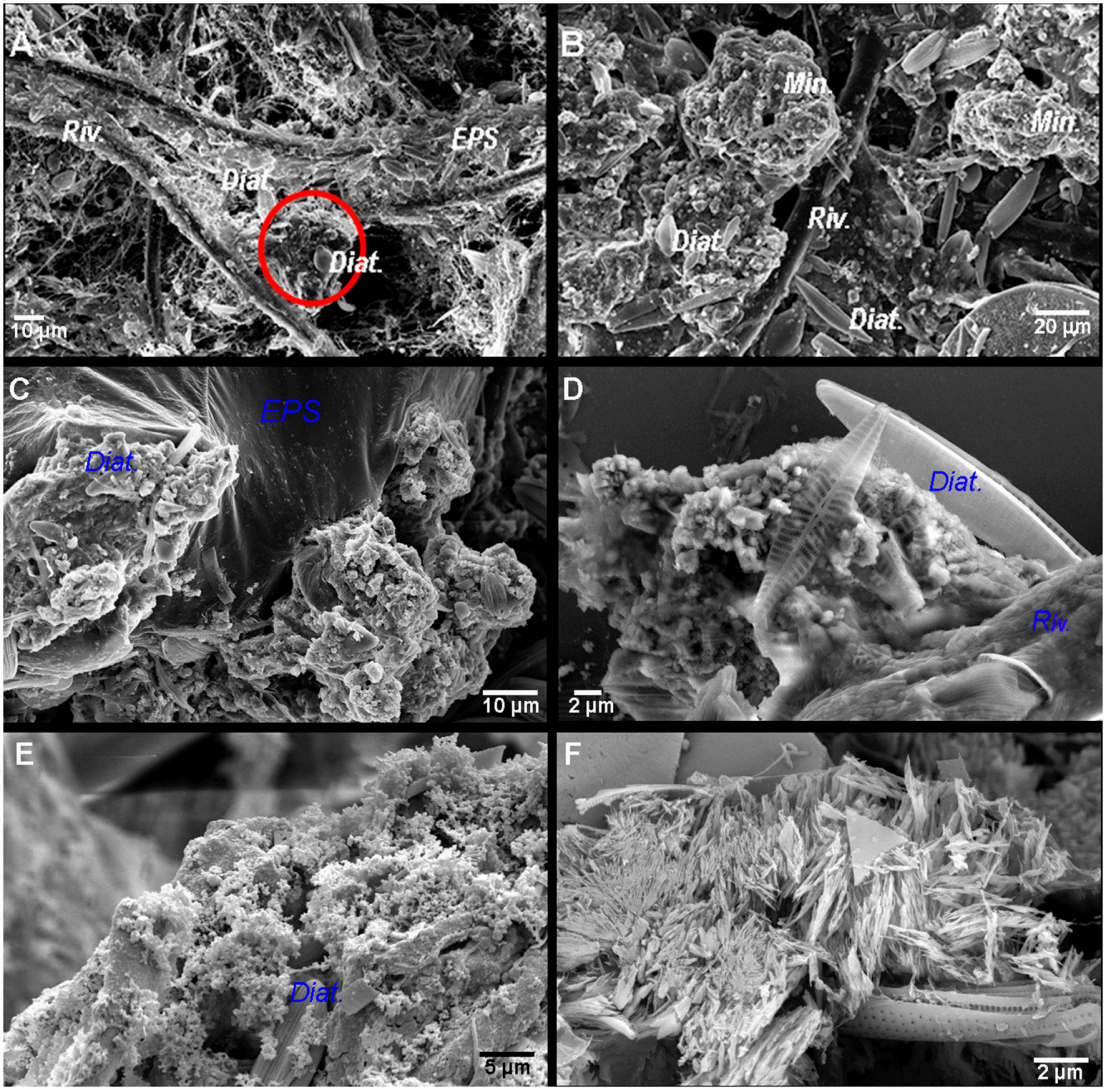
**(A)** SEM images of the surface of the black pustular mat where *Rivularia* filaments (*Riv*.) and exopolymeric substances (*EPS*) are observed. The red circle highlights a mineral aggregate with diatoms (*Diat*.). **(B)** Below the black pustular surface, mineral aggregates (*Min*.) with diatoms are abundant; some *Rivularia* filaments can also be observed. From **(C–D)**, SEM images in backscattered electron mode of the carbonate minerals associated with the *Rivularia* filaments in BP mats. **(C)** Aggregates formed by sub-spherical to subhedral carbonate particles associated with the EPS matrix where diatoms frustules are also observed. **(D)** Magnified view of a mineral aggregate with subspherical carbonate grains and associated diatoms. **(E)** Aggregate composed of submicrometric carbonate spherules entombing abundant diatoms frustules. **(F)** Elongated needle-like carbonates.

Most of the *Rivularia* filaments were observed in the superficial pustular zone of the BP mats. They showed a tangled arrangement with variable amounts of EPS-associated mineral aggregates and diatoms located between the cyanobacterial filaments (Figure 6A). The lower (i.e., inner) part of the BP mats showed *Rivularia* filaments together with calcium carbonate precipitates within an EPS matrix and diatoms (Figure 6B). Locally and within the carbonates, some tubular hollow structures were observed (Figure 6B). They probably corresponded to entombed microbial filaments. The diameter of these hollow structures was variable, being similar or lower than that of fresh *Rivularia* filaments (Figure 6B).

XRD analyses of bulk samples of the BP mats showed that they were mainly composed of Mg-calcite, with a lower amount of aragonite (Supplementary Figure S1). At the microscale, as observed using SEM, different types of carbonate particles were identified, with the predominance of sub-spherical to subhedral carbonate particles with diameters varying from 80 to 700 nm (Figure 6C) along with almost perfect tiny carbonate spheres (Figure 6E). Occasionally more irregular anhedral carbonate particles of similar size were also observed (Figure 6D). Sub-spherical to subhedral particles were typically associated with EPS and sometimes clustered into irregularly shaped aggregates of various sizes (usually up to ~200 μm in diameter). These aggregates were closely associated to the EPS matrix where abundant diatoms and other microorganisms were also present (Figures 6C–F). They occasionally clumped together and form wavy to irregularly shaped horizontal lamina.

Another distinct group of calcium carbonate particles was represented by irregular bunches of elongated, needle- and spindle-like particles (Figure 6F). The individual needle-shaped particles could be up to 2 μm long and formed irregularly distributed clusters or patches that were not typically associated with the EPS matrix. As suggested by XRD data and given the acicular shape these particles, these needles could be aragonite. While the precipitation of the nanometer-sized sub-spherical to spherical calcium carbonate grains appeared to be limited to the EPS matrix of the top-most layers of the mat, the larger subhedral and euhedral grains were mostly present in the inner parts of the mat, where degraded diatoms and cyanobacterial sheaths were present.

#### Calcein staining of resin-embedded BP mat: calcium localization

**Figure 7:**
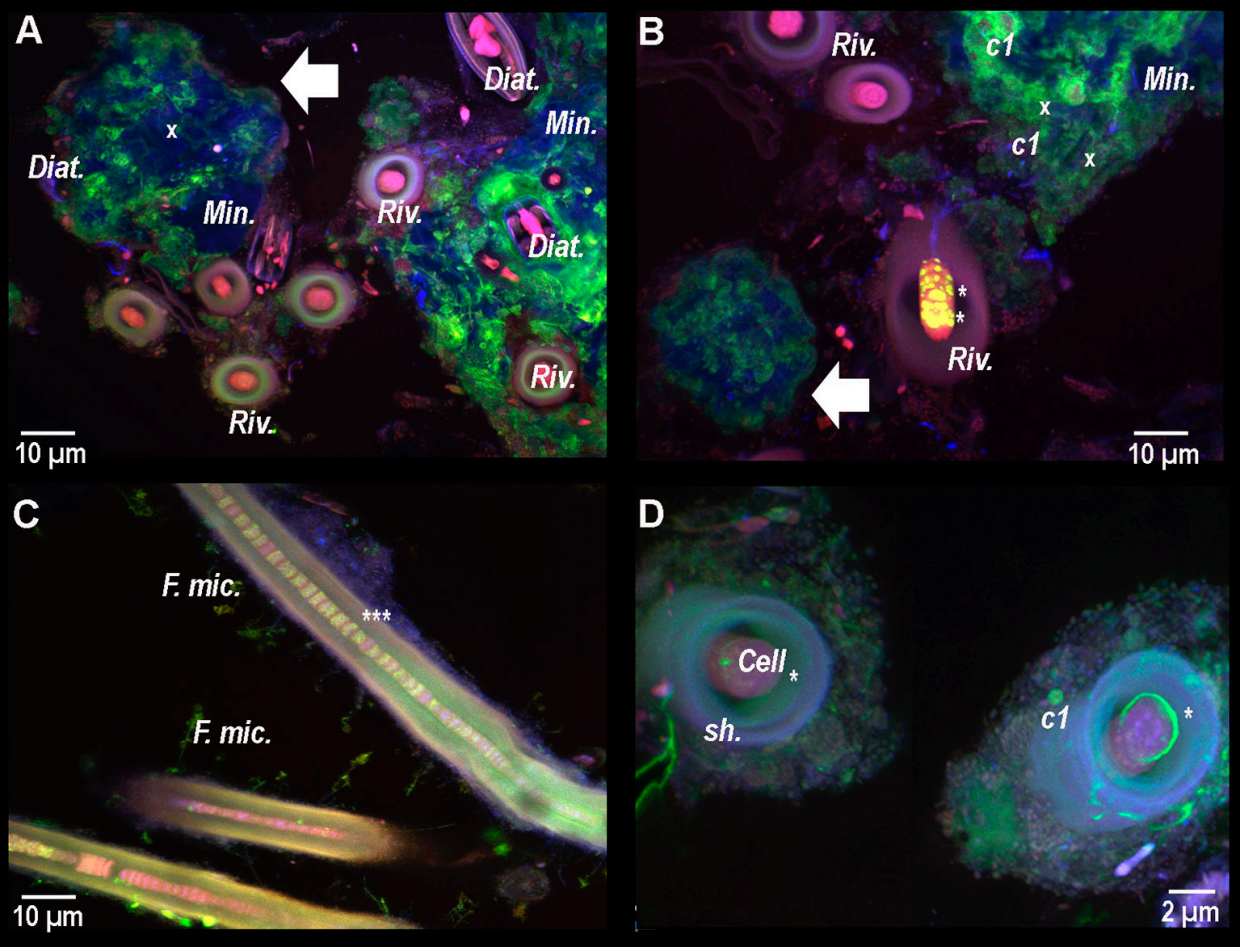
Composite CLSM images of a resin-embedded black pustular microbialite, stained with calcein. Images were obtained with concomitant excitations at 405, 488, and 543 nm and collection between 425 and 475 nm, 500 and 530 nm, and 560 and 660 nm, respectively. Fluorescence emission between 500 and 530 nm emerges specifically from calcein (in green). **(A)** Mineral aggregates (*Min*. and white arrow) are observed in blue. Their surfaces are partially stained with calcein, hence indicating the presence of free Ca^2+^ ion. Inside and surrounding the mineral aggregate, diatom frustules (x) and living diatoms (*Diat*.) are visible thanks to their photosynthetic pigments. Some *Rivularia* filaments (*Riv*.) seem to be separated from the mineralized aggregates, while other filaments are close but not entombed in the aggregate and occur with diatoms. Calcein stained the space inside the filaments, i.e., between the sheath and the cells. In **(B)** yellow/light green dots are observed in one of the *Rivularia* filament (^*^). Calcein also strongly stained the contours of the pigmented *c1* cocci-shaped cell colonies that were found closely associated with the mineral aggregates (white arrow). **(C)** Close up view of calcein-stained *Rivularia* filaments. The dye also stained some other filamentous microorganisms associated with the *Rivularia* sheath (*F. mic*.). Inside the *Rivularia* filament, some green dots are distinguishable (^***^). **(D)** Image of two transversally-cut *Rivularia* filaments, where calcein fluorescence (^*^) was also found between the cells and the sheath (*sh*.); as in **(C)**, calcein also stained the *c1* colonies.

In order to study the micro-scale relationships between microorganisms and carbonate precipitates within the consortium, resin-embedded microbial mat fragments stained with calcein were analyzed by CLSM. Calcein stained the active parts of mats displaying free divalent cations close to the minerals and the subspherical aggregates located in between the *Rivularia* filaments (Figures 7A,B). Besides, the cocci cells (*c1*) already observed in Figure 3, were also visible with abundant calcein staining around the colonies (Figure 7B), all in close spatial relationship with the *Rivularia* sheaths (Figure 7D). Interestingly, calcein accumulated in between the *Rivularia* sheaths and cells (Figures 7A–D). Some bright spots were also detected at this level (Figures 7B–D), suggesting a possible mechanism of calcium accumulation or trafficking. As only Ca^2+^ was detectable using SEM-EDXS around the cells stained with calcein (Supplementary Figure S2), this calcein coloration should mainly reveal Ca^2+^ accumulation rather than Mg^2+^ accumulation.

#### Transmission electron microscopy observations of a cyanobacterial filament

In order to explore the possibility of calcium accumulation inside the filaments, as suggested by the bright spots observed in Figures 7B–D, FIB ultrathin sections were milled from resin-embedded BP mat samples showing calcein-stained *Rivularia* filaments. This procedure allowed exploring the free Ca and Mg content of cyanobacterial sheaths without any interference coming from the surrounding minerals.

**Figure 8:**
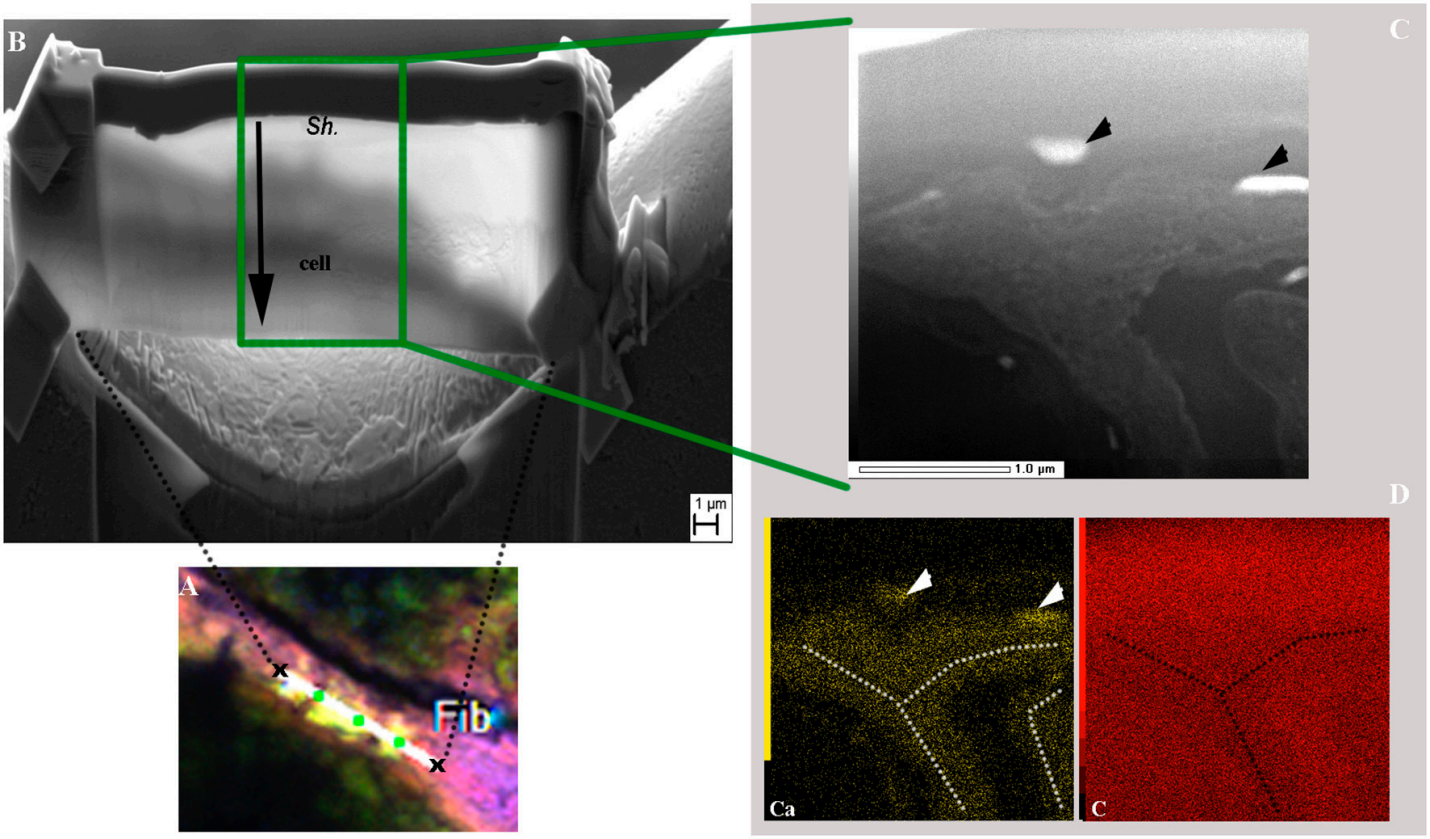
SEM and TEM observations of a FIB section longitudinally milled on a cyanobacteria filament stained with calcein as shown in the CLSM image displayed in **(A)** (see Supplementary Figure S3 for location of the selected area and associated scale). **(B)** SEM image of the ultrathin section after milling. The upper part of the section corresponds to the filament sheath (*Sh*.) and the lower part (end of black arrow) to the cyanobacteria cell. **(C)** HAADF-STEM image of the FIB section showing the filament along its longer axis and the different phases within the filament. The black arrowheads indicate regions with strong electron absorption. **(D)** Associated STEM-EDXS elemental maps for calcium (Ca; in yellow) and carbon (**C**; in red) displaying opposite distributions. Ca-rich areas are highlighted by white arrowheads and white dotted lines.

A longitudinal *Rivularia* filament stained with calcein was selected with others for FIB milling after CLSM observation (Supplementary Figure S3 and Figure 8A) and transversally milled (Figure 8B). HAADF-STEM observation of the FIB section with associated STEM-EDXS mapping of calcium (Ca, yellow) and carbon (C, red), is shown in Figures 8C,D. These two elements exhibited opposite distributions. Calcium-rich areas (white and black dotted lines) corresponded to low-carbon areas. Localized calcium globules were also detected (black and white arrows in Figures 8C,D, respectively). No clear or very low Mg signal was detected in the analyzed samples, supporting the idea of Ca accumulation within the filaments. These observations were consistent with CLSM observations showing high calcein fluorescence between the *Rivularia* sheaths and the cells. Nevertheless, in this case no crystalline phase was detected inside the *Rivularia* sheaths or cells.

### Characterization of the structure and mineralogy of *Rivularia*-rich laminations in oncoids associated with the BP mats

**Figure 9:**
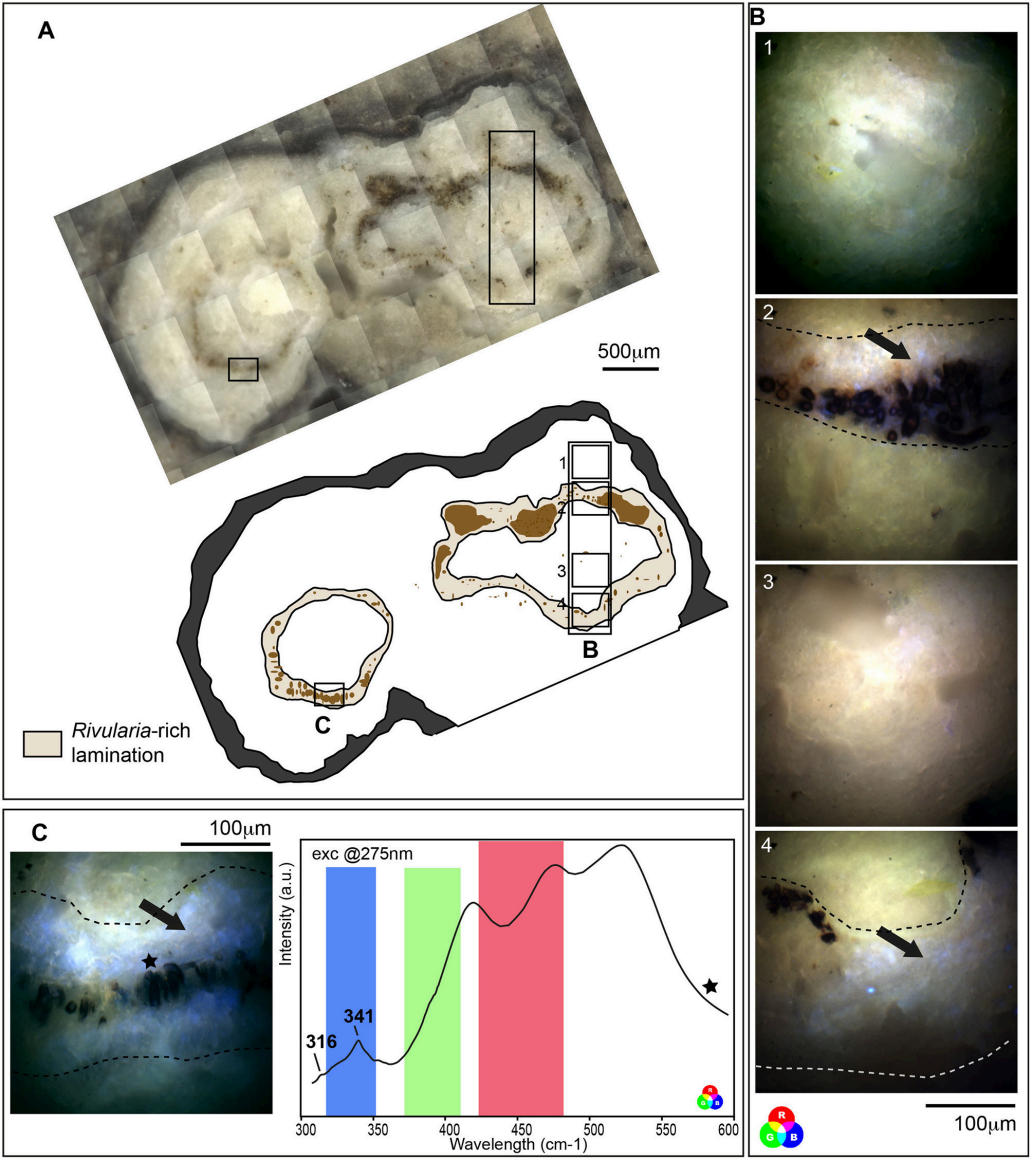
Fluorescence emission signal after synchrotron-based deep UV excitation at 275 nm of a cross section of an oncoid associated with a black pustular mat. **(A)** Optical microscopy observation and schematic representation of the cross section highlighting *Rivularia*-rich laminations with encrusted *Rivularia*-like cells. The localization of areas of interest analyzed by S-DUV fluorescence imaging is given. **(B)** Composite RGB images of 4 areas of interest were reconstructed using the fluorescence signal collected with filters between 327 and 353 nm (Blue), 370 and 410 nm (Green) and 420 and 480 nm (Red). They were surimposed on the optical image. An intense fluorescence signal was observed using the blue filter between 327 and 353 nm (black arrows) in *Rivularia*-rich laminations (underlined by dot lines in **B,C**) while the rest of the matrix is marked by a mixed fluorescence collected using the green and red filters. **(C)** The fluorescence emission signal associated with *Rivularia*-rich laminations was recorded and showed two bands at 316 and 341 cm^−1^. The fluorescence ranges covered by the three filters are indicated with respective colors and the precise localization where the spectrum was collected is given by a black star on the associated full-field RGB fluorescence image.

SEM and CLSM observations indicated that, in the mat, carbonate precipitation seemed to occur within the EPS excreted by the microbial consortium found associated with the *Rivularia* filaments, which dominate the black pustular mat. The corresponding laminations within the associated oncoids should then be considered as biologically-induced and may have then registered direct or indirect traces of biological activity. We then investigated these laminations in oncoids in order to characterize potential biomarkers to be searched for in fossil stromatolites. Synchrotron-based deep UV fluorescence imaging was first carried out on a cross-section from an oncoid associated with the BP mats collected in 2013 (Figure 9). Optical microscopy observations of this cross section highlighted the presence of different laminations presenting variable colors (Figure 9A). Among them, brown-beige laminations were clearly distinguishable from other ones and were marked by the presence of numerous encrusted *Rivularia*-like filaments (Figure 9A). Full-field fluorescence images were collected on a transect crossing *Rivularia*-rich laminations (Figure 9B) after excitation at 275 nm. Similar imaging was performed on other *Rivularia*-rich laminations (Figure 9C) for consistency. Reconstructed RGB images indicated that the fluorescence signal was different in these laminations compared to the bulk carbonated matrix of the oncoid (in white in Figure 9A). These laminations (Figure 9B images 2 and 4, and Figure 9C) display fluorescence emission in the range 327–357 nm (detected with the Blue filter) while white laminations were dominated by a mix of fluorescence emission signals collected with the green and red filters (leading to a yellow color; Figures 9B,C). These observations were consistent for the whole sample and indicated that *Rivularia*-rich laminations presented a different structure or composition compared to the other laminations. In order to decipher the origin of this specific signal, hyperspectral fluorescence images were acquired on the same sample. An example of the collected fluorescence emission signal is given Figure 9C. Spectra were characterized by two bands at 316 and 341 nm and a large massif above 400 nm. The latter one could not be properly deconvoluted due to optical artifacts occurring above 400 nm. The two other bands at 316 and 341 nm were likely emerging from organic compounds related to cell remnants (main fluorescence emission at 312, 340, and 410 nm; Jamme et al., 2013) trapped in the mineralized matrix. The difference in fluorescence emission signals observed between *Rivularia*-rich laminations and other laminations could then correspond to a different content in organic compounds. However, carbonate minerals are known to fluoresce when excited in the UV range. The variable fluorescence patterns from one lamination to another one might then also be induced by differences in the lamination mineralogy.

**Figure 10:**
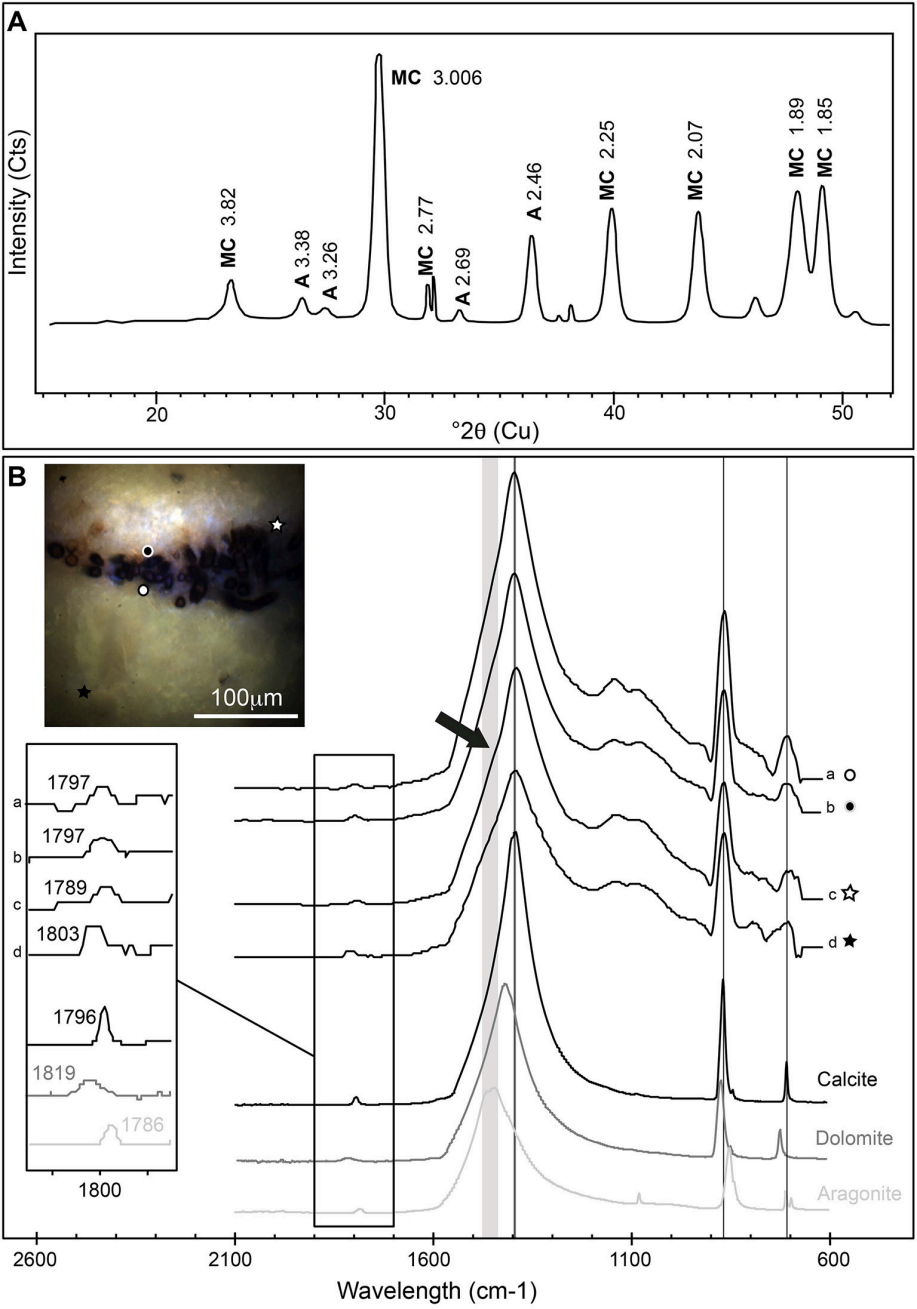
**(A)** X-ray powder diffractogram obtained on a *Rivularia*-rich lamination. It shows Mg-rich calcite (MC) and aragonite **(A)** (Cts stands for counts). **(B)** ATR-FTIR spectra obtained on a *Rivularia*-rich lamination from a cross section of an oncoid associated with a black pustular mat also analyzed by S-DUV (Figure 9B). The precise localization of the spectra is given in the associated full-field RGB fluorescence emission image (white and black circles and stars). ATR-FTIR spectra were compared to standard spectra of calcite, aragonite and dolomite (Rruff database; http://rruff.info/) and showed the predominance of calcite mixed with either a Mg-carbonate or aragonite, detected by the shift of the CO_3_^2−^ vibration bands at 1,796 cm^−1^ and a shoulder on the CO_3_^2−^ asymmetric stretching band around 1,400 cm^−1^ (black arrow).

The mineralogy of *Rivularia*-rich laminations was then investigated. Bulk XRD analyses were first performed on powdered oncoid associated with BP mat (Figure 10A) and indicated the predominance of Mg-rich calcite (90%) associated with minor aragonite (10%). Microscale ATR-FTIR analyses were then performed on the cross-section analyzed by S-DUV fluorescence imaging, specifically in the areas presenting a “blue” fluorescence pattern (Figure 9B images 2 and 4, and Figure 9C). Most of the acquired ATR-FTIR spectra (*n* ≈ 66) presented a similar pattern and are summarized in Figure 10B. The position of main CO_3_^2−^ vibrational bands (ν_2_ asymmetric bending at 870 cm^−1^; ν_3_ asymmetric stretching at 1,400 cm^−1^; ν_1_ + ν_4_ at 1,800 cm^−1^) were compared to carbonate standards and mainly corresponded to calcite (e.g., spectra a and b in Figure 10B). Some spectra presented patterns that might indicate a mixed composition between calcite and other carbonates depending on the position in the lamination. The low spatial resolution of the ATR tip (20 × 20 μm) precluded the identification of individualized pure components at the microscale. However, some spectra acquired very close to *Rivularia*-like cell remnants presented shifts from the typical calcite ν_2_ asymmetric bending band at 1,789 cm^−1^ (vs. 1,796 cm^−1^ for calcite), along with a marked shoulder in the ν_3_ asymmetric stretching band (black arrow in spectrum c displayed in Figure 10B). It should correspond to a mix between calcite and aragonite. Similarly, analyses in a white lamination, far from the assumed influence of *Rivularia*-rich lamination, presented a shift of the ν_2_ asymmetric bending toward 1,803 cm^−1^. It may highlight a mixed composition between calcite and dolomite (spectrum d in Figure 10B) and should be indicative of the presence of Mg-bearing carbonate, consistently with bulk XRD analyses. These combined analyses seem to confirm that the *Rivularia*-rich laminations should be distinguishable from the other ones from a spectroscopic point of view. Even if we cannot non-ambiguously conclude, they may present a higher organic content than other laminations (without *Rivularia* filaments) and/or a different mineralogy.

## Discussion

The different microscopic observations and microbial diversity analyses carried out in this study highlight the likely influence of the microbial consortium observed around *Rivularia* filaments in the carbonate precipitation process. Thus, the role of *Rivularia* and of the related consortium in the mineralization processes is discussed. In parallel, the composition of the corresponding laminations in oncoids, comparatively to other laminations, is discussed in order to characterize potential biosignatures to be searched for in fossil stromatolites.

The most abundantly detected cyanobacterial 16S rRNA gene sequences using the cyanobacterial specific primer were affiliated to the *Phormidium* and *Rivularia* genus. The large filamentous cyanobacteria of around 15 μm in diameter, observed by optical microscopy and CLSM, were identified as *Rivularia* using laser microdissection (Table 1, Figure 4). A striking observation is that calcein stained the space between the cells and the sheath of the *Rivularia* filaments (Figure 7). It suggests a possible mechanism of Ca^2+^ concentration and may have implications for carbonate precipitation. This could be due to the difference between the cell wall and the sheath surface that was observed for *Calothrix* sp. (strain KC97; Phoenix et al., 2002), a cyanobacteria presenting a morphology similar to the one of the *Rivularia* sp. *Calothrix* sp. has a spatially dual-layer system composed of a reactive cell wall, and a poorly-reactive sheath. The sheath may then inhibit detrimental biomineralization or HCO_3_^−^ diffusion. On the contrary, the cell wall, which contains a high density of electronegative sites, may trap cations (Phoenix et al., 2000, 2002). A similar process should be proposed for the *Rivularia* of Laguna Negra. It would explain the specific accumulation of Ca^2+^ observed close to the cell wall surface rather than on the external sheath itself. Conversely, it has been shown that living cyanobacterial cells are capable of self-protection against solid carbonate incrustation through shedding of mineralized S-layer (Thompson et al., 1997; Douglas and Beveridge, 1998) and/or by metabolically maintaining positive surface potential to avoid Ca^2+^ adsorption and subsequent entombment within solid carbonates (Martinez et al., 2008, 2010). On the other hand, in cyanobacteria, as for all organisms, intracellular calcium is strongly regulated by independent processes of Ca^2+^ uptake and active efflux (Smith and Wilkins, 1988). The normal levels of Ca^2+^ are maintained very low in order to prevent toxicity. If external Ca^2+^ concentrations are higher, as it is typically the case in hypersaline lakes, calcium uptake may involve low passive permeability of Ca^2+^ sensitive trans-membrane channels (Singh and Mishra, 2014). In the case of the Andean *Rivularia* sp. identified in this study, further efforts should be devoted to understand why *Rivularia* filaments are capable to keep free Ca^2+^ ions between the cells and the sheaths without inducing carbonate precipitation, and if this calcium could bind to the S-layer domains (e.g., glutamate and aspartate residues) or to the cell wall domains to control the flux of calcium in and out of cells.

Despite the alkalinizing activity of *Rivularia* cyanobacteria and contrary to what was described for freshwater *Rivularia* (Pentecost and Ulrich, 2010), a remarkable observation of the present study is that the *Rivularia* filaments did not present any carbonate precipitation close to their sheath (Figures 3, 6, 7). Calcification in cyanobacteria depends on local environmental conditions (Arp et al., 2001; Riding, 2006) and only occurs in waters supersaturated with respect to calcium carbonates. However, only certain genera calcify and none of them are obligate calcifiers (Merz, 1992). The microorganisms located on the cyanobacterial sheath may metabolically modify the local physico-chemical conditions and induce or preclude carbonate precipitation. We assessed the influence of the activity of epiphytic bacteria on carbonation, based on the results obtained by 16S rRNA encoding genes sequencing following laser microdissection and whole genome amplification. Most of the bacteria associated with the *Rivularia* sheaths were affiliated to known epiphytic members of the Bacteroidetes phylum. Some identified species were affiliated to the *Maribacter* genus that encompasses heterotrophic bacteria associated with brown algae (Nedashkovskaya et al., 2004). Their activity may accordingly induce acidic conditions by producing CO_2_ around *Rivularia* sheaths, hence precluding carbonate precipitation (Dupraz and Visscher, 2005; Dupraz et al., 2009) and explaining the absence of carbonation on *Rivularia* sheaths in Laguna Negra.

On the contrary, the results obtained in this study indicated that carbonate precipitation occurred within the EPS matrix excreted by the diverse bacterial consortium associated with *Rivularia*. To identify potential calcifiers in the BP mats, the influence of the whole microbial community retrieved by bulk phylogenetic analyses was then assessed in light of microscopic observations. SEM and CLSM observations indicated that calcification might start with the precipitation of small isolated granules of calcium carbonates dispersed throughout the EPS matrix surrounding diatoms and coccoid pigmented cells (*c1*) (Figures 3, 7) but not in direct contact with the *Rivularia* sheath. Diatoms and other microorganisms such as Myxococcales and methanogenic archaea are known to produce large amounts of EPS (Bapteste et al., 2005; Scholten et al., 2005). These EPS should serve as nucleation sites for carbonate precipitation following organo-mineralization processes (Perry et al., 2007; Defarge et al., 2009). This is consistent with the micritic anhedral globular textures observed by SEM, characteristic of carbonate precipitation occuring in an organic matrix (Dupraz et al., 2009). The primary amorphous grains would be bound to EPS surface charges and would then grow into larger subhedral to euhedral carbonate grains. The precipitation of these carbonates would be favored by the supersaturation of the waters relatively to calcite (Gomez et al., 2014) and by some local alkalinization induced by microbial activity. Notably, in addition to the photosynthetic activity of *Rivularia*, diatoms may also have promoted local alkalinization. Furthermore, some of the bacteria identified in the whole microbial community may present potential alkalinizing metabolisms favoring carbonate precipitation. Among them are the Myxococcales identified in the BP mats of Laguna Negra. Some bacteria belonging to this order are known to favor mineral precipitation (Jimenez-López et al., 2007), such as *Myxococcus xanthus*, which induces carbonate precipitation by ammonification, resulting in an increase of the alkalinity in the culture medium (González-Muñoz et al., 2010). Of likely greater interest, the pigmented coccoid bacteria (*c1*, Figure 3) may correspond to some of the aerobic anoxygenic phototrophic bacteria (AAnPB) affiliated to the marine *Roseobacter* clade (Figure 5 and Table 2). Abundant and diverse AAnPB were detected in the BP mats (Table 2). The phylogenetic analysis of the 16S rRNA and *pufLM* encoding genes showed that specific AAnPB develop in hypersaline high altitude Andean lakes. Members of the *Roseobacter* clade are known to interact with marine phytoplankton, including diatoms, also abundant in Andean lakes (Maidana and Seeligmann, 2006; Farías et al., 2013, 2014; Barbieri et al., 2014). This association may allow microbes to use metabolic niches that would be inaccessible otherwise (Overmann and van Gemerden, 2000; Schink, 2002; Orphan et al., 2008). For example, *Roseobacter-*related species are able to use glycolate excreted by eukaryotic phytoplankton during autotrophic photorespiration (Fogg, 1983; Grossart et al., 2005). Members of the *Roseobacter* clade are phototrophic bacteria but lack genes for inorganic carbon fixation (Lenk et al., 2012; Luo and Moran, 2014; Zhang et al., 2016). Consequently, these bacteria cannot favor carbonate precipitation *via* alkalinization linked to photoautotrophy (Dupraz and Visscher, 2005). On the contrary, most of the members of the *Roseobacter* clade are ureolysers and some are denitrifyers (Luo and Moran, 2014). Both ureolysis (Zhu and Dittrich, 2016) and denitrification (Erşan et al., 2015) increase pH in the surrounding medium and favor carbonate precipitation. Furthermore, it has been shown that denitrification activity of the *Roseobacter denitrificans* strain was increased by light stimulus (Doi and Shioi, 1991). Even if we cannot preclude the influence on carbonate formation of other microbial strains, such as methanogenic archaea (Michaelis et al., 2002; Roberts et al., 2004; Scholten et al., 2005; Kenward et al., 2009), we suggest that phototrophy, ureolysis and denitrification associated with the activity of AAnPB may be important drivers of alkalinization and carbonate precipitation in the BP mats of Laguna Negra.

Overall, *Rivularia* cyanobacteria favored the development of a large microbial consortium that indirectly promoted carbonate precipitation via organo-mineralization processes, and hence participated to the formation of stromatolites. The corresponding laminations in oncoids should then be considered as biologically-induced. They may present some direct or indirect markers of biological activity or cell remnants that may furnish biosignatures that could be searched for in the geological record. We then investigated stromatolites associated to the black pustular mat at Laguna Negra. We focused on laminations that presented *Rivularia*-like encrusted cells and we compared them to micritic and botryoidal laminations of the stromatolites, which may have a purely chemical origin. The laminations of interest presented well-preserved filaments that may have been encrusted *in vivo* or rapidly after death while preserving the original sheath morphology and sizes (Riding, 1977; Merz-Prei and Riding, 1999; Couradeau et al., 2014). The morphology of the mat could indeed be preserved if the carbonate formation process is fast enough to cover the EPS (Kazmierczak et al., 2015), hence creating mineral coatings on cells or groups of cells. Besides, these encrusted filaments presented a strong fluorescence signal after CLSM that could be attributed to cell photosynthetic pigments (Figure 2D). Pigments are recalcitrant molecules that can be preserved in sediments (Leavitt et al., 1997). They could be used as molecular fossils of photosynthetic organisms (Brocks and Pearson, 2005). The analysis of the *Rivularia*-rich laminations of oncoids associated with BP mats using S-DUV also clearly highlighted a different fluorescence signal compared to other laminations after excitation in the deep UV range (Figure 9). The origin of the fluorescence pattern could not be fully elucidated but could be attributed either to (i) a different mineralogy or (ii) a higher content in organic compounds trapped in the mineral matrix relatively to other laminations. Punctual FTIR analyses of the *Rivularia*-like laminations seemed to indicate a possible higher proportion of aragonite relatively to calcite or Mg-calcite in these areas compared to other laminations. This could partly explain the different fluorescence pattern of this lamination. In that case, the presence of aragonite-rich laminations relatively to calcite-rich laminations may be a clue of biological activity (Lepot et al., 2008). Besides, the presence in the deep UV fluorescence emission spectrum extracted from this lamination (Figure 9C) of typical peaks associated with biological organic remnants at 316 and 341 cm^−1^ (Jamme et al., 2013) seemed to confirm the biological influence on these laminations. On the contrary, the absence of these signals in the other laminations may indicate that micritic and botryoidal laminations were either of pure chemical origin or did not preserve biological organic remnants if they were initially present. In the latter case, we may postulate that the presence of *Rivularia* sheaths, which favor the development of a large microbial consortium and are quite resistant to degradation, may have induced the preservation of a larger amount of biological organic remnants during mineralization comparatively to other laminations. It has important consequences for the search for biological traces in the fossil record. Indeed, these results are consistent with Lepot et al. (2008) who assumed a biological origin for stromatolites from the 2,724-Myr-old Tumbiana formation (Australia) based on the association of aragonite nanocrystals with organic globules in these formations. Specific laminations such as the ones rich in *Rivularia*-like structures presenting both higher aragonite together with a high organic content should then be tracked in order to decipher the biological origin of some laminations in fossil stromatolites.

## Conclusion

By combining microscopic observations jointly with phylogenetic analyses, this study provides potential pathways for carbonate precipitation in the black pustular mat at Laguna Negra. Mineralization does not initiate directly on the *Rivularia* sheath or in the *Rivularia* sheath-cell interspace. This could be respectively related to the activity of epiphytic bacteria and to the capacity of *Rivularia* to locally change calcium concentrations by shedding a possible mineralized S-layer or by maintaining a positive surface potential to avoid Ca^2+^ adsorption. However, *Rivularia* play a critical role in favoring and structuring the development of a large microbial consortium excreting a well-developed EPS matrix. Carbonate precipitation then occurred *via* organo-mineralization processes, the EPS matrix serving as a template for mineral nucleation. In addition to the alkalinizing activity of *Rivularia* and diatoms, the presence of putative AAnPB of the *Roseobacter* clade suggests that ureolysis and denitrification can be important metabolisms triggering carbonate precipitation by favoring local alkalinization. With progressive precipitation, *Rivularia* get entombed with intact sheath and photosynthetic pigments. The microspectroscopic analyses of the corresponding laminations rich in *Rivularia*-sheaths structures in stromatolites indicate that a close combination of aragonite and a high organic content should then be considered as potential evidence of microbially-mediated processes of formation of stromatolites, providing biosignatures to be searched for in the fossil record.

## Author contributions

EG, EM, and CP were responsible for the design of the study. EG, EM, CP, BM, LL, and FG performed experimental procedures and collected data. EG, KB, FG, and EM conducted FIB section, SEM and TEM. EG, EM, and LL performed DNA extraction and PCR. CP and EG performed FTIR analyses; CP, EG, BM, EM, FJ, and MR performed synchrotron-based Deep UV imaging experiment. ES performed XRD analyses. EM, CP, and EG interpreted the findings and drafted the manuscript. All authors critically reviewed content and approved the final version for publication.

### Conflict of interest statement

The authors declare that the research was conducted in the absence of any commercial or financial relationships that could be construed as a potential conflict of interest.
