# Supplementary material for: Characterization of Pustular Mats and Related Rivularia-Rich Laminations in Oncoids From the Laguna Negra Lake (Argentina)
Source: Front Microbiol. 2018 May 22;9:996. doi: 10.3389/fmicb.2018.00996 (PMC5972317; doi:10.3389/fmicb.2018.00996)
Supplement: Supplementary file 1 [file Data_Sheet_1.PDF]

Supplementary Information

Characterization of pustular mats and related *Rivularia*-rich laminations in oncoids from the Laguna Negra lake (Argentina)

Estela C Mlewski[1*#], Céline Pisapia[2,3#], Fernando Gomez[1], Lena Lecourt[2], Eliana Soto Rueda[1], Karim Benzerara[4], Bénédicte Ménez[2], Stephan Borensztajn[2], Frédéric Jamme[3], Matthieu Réfrégiers[3], Emmanuelle Gérard[2]

[1] Instituto de Investigación en Ciencias de la Tierra, CICTERRA-UNC-CONICET, Córdoba, Argentina

[2] Institut de Physique du Globe de Paris, Sorbonne Paris Cité, Université Paris Diderot, CNRS, Paris, France

[3] Synchrotron SOLEIL, DISCO beamline, Saint Aubin, France

[4] Institut de Minéralogie, de Physique des Matériaux et de Cosmochimie, UMR CNRS 7590, Sorbonne Université, Muséum National d'Histoire Naturelle, IRD UMR 206, Paris, France

*** Corresponding author:**
Estela Cecilia Mlewski, Ph.D.
Laboratorio de Geomicrobiologia, CICTERRA-CONICET, Universidad Nacional de Córdoba, Av. Velez Sarsfield 1611, 5016 - Córdoba Argentina.
Phone: + 54-0351-4344980 (ext. 116)
E-mail: cmlewski@gmail.com

Emmanuelle Gérard, Ph.D.
Institut de Physique du Globe de Paris, Sorbonne Paris Cité, Université Paris Diderot, CNRS, Paris, France.
Phone: + 33(0)183957383
E-mail: emgerard@ipgp.fr

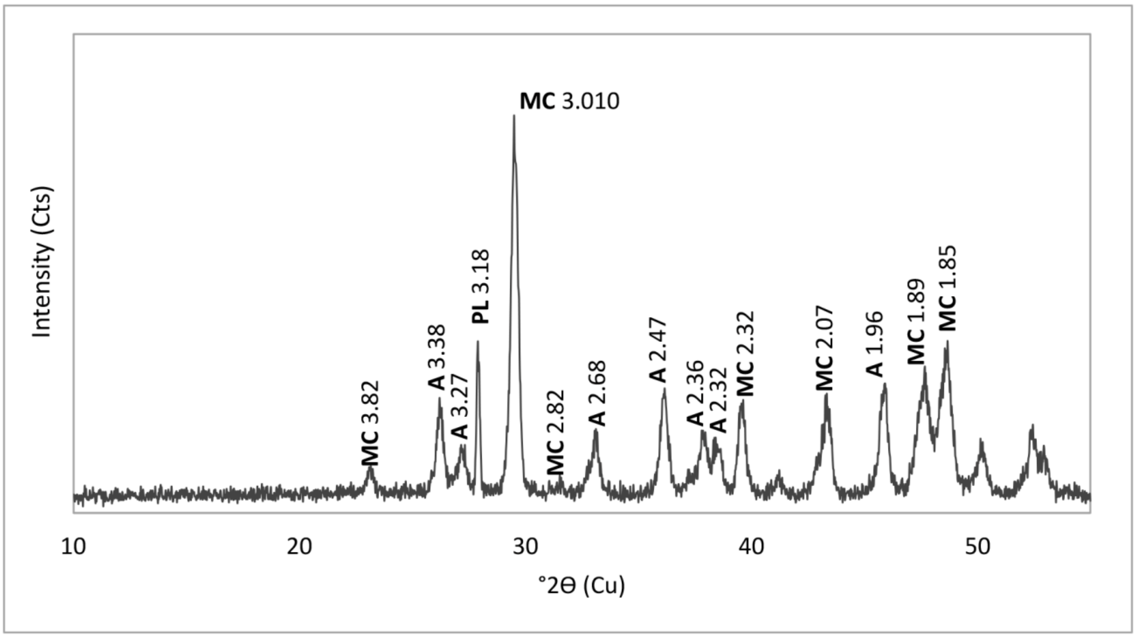

Supplementary Figure S1: X-ray powder diffractogram obtained on the BP mat showing mainly Mg-rich calcite (MC), aragonite (A) as well as few plagioclases (PL). “Cts” stands for counts.

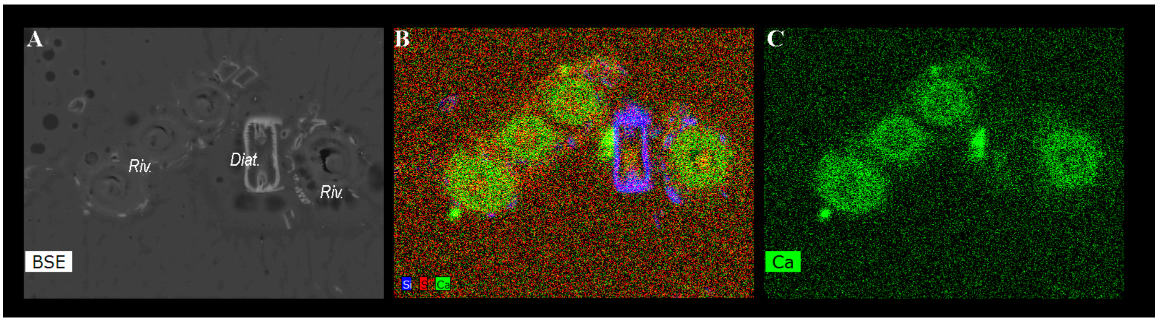

Supplementary Figure S2: (A) SEM image in backscattered electron mode of a cross-section of resin-embedded BP mat showing *Rivularia* filaments (*Riv.*) close to diatoms (*Diat.*). **(B)** Associated composite EDS map merging Ca (in green), Si (in blue), and S (in red). Ca distribution is presented individually in **(C).** It shows preferential accumulations of Ca in the *Rivularia* filaments compared to its surrounding.

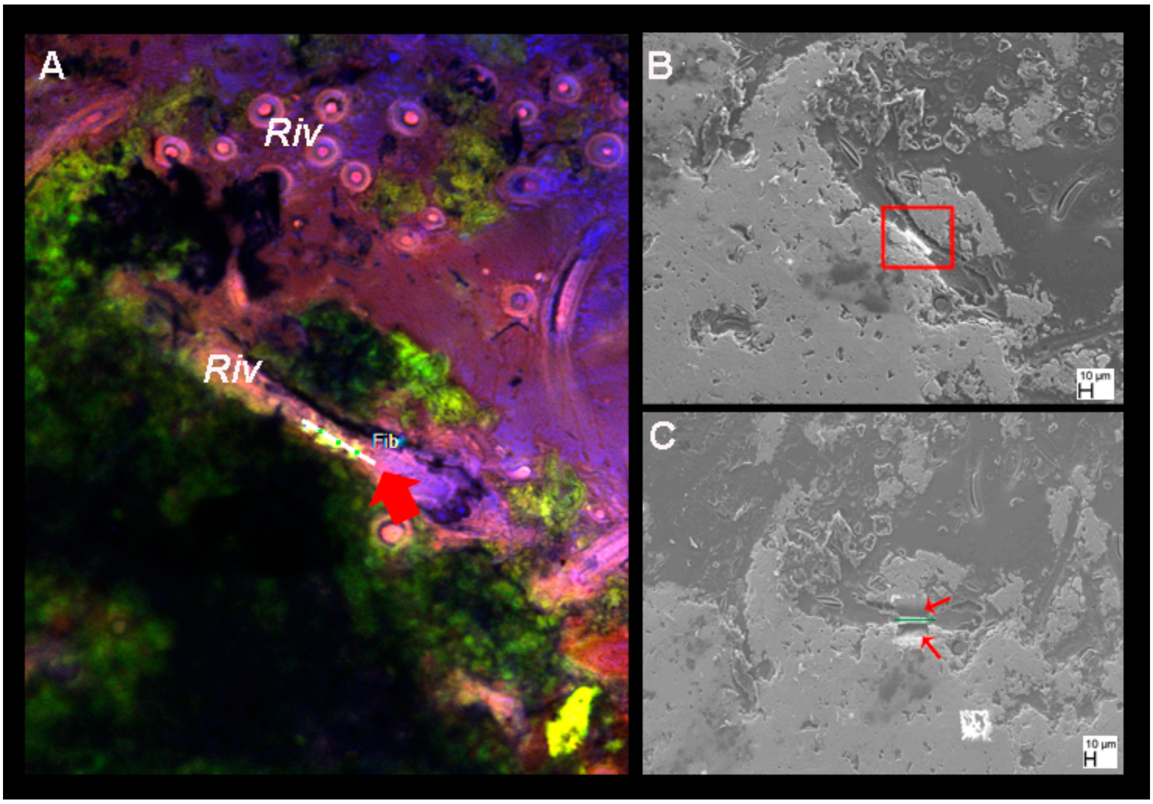

Supplementary Figure S3: (A) CLSM image of a resin-embedded BP mat stained with Syto®9 (green) and highlighting free Ca in the surrounding of the *Rivularia* filaments (*Riv*). Red arrow shows the calcein-stained place between the sheath and the cell where a platinum coating was deposited for FIB milling. **(B)** SEM image showing the platinum (in the middle of the red square) already deposited on the filament. **(C)** SEM image showing the orientation of the milled area (green line) with excavations on both sides indicated by red arrows.
